# Review of Artificial Intelligence Techniques for Breast Cancer Detection with Different Modalities: Mammography, Ultrasound, and Thermography Images

**DOI:** 10.3390/bioengineering12101110

**Published:** 2025-10-15

**Authors:** Aigerim Mashekova, Michael Yong Zhao, Vasilios Zarikas, Olzhas Mukhmetov, Nurduman Aidossov, Eddie Yin Kwee Ng, Dongming Wei, Madina Shapatova

**Affiliations:** 1 School of Engineering and Digital Sciences, Nazarbayev University, Astana 010000, Kazakhstan; aigerim.mashekova@nu.edu.kz (A.M.); yong.zhao@nu.edu.kz (M.Y.Z.); olzhas.mukhmetov@nu.edu.kz (O.M.); nurduman.aidossov@nu.edu.kz (N.A.); 2 Department of Mathematics, University of Thessaly, 35100 Lamia, Greece; vzarikas@gmail.com; 3 Mathematical Sciences Research Laboratory (MSRL), 35100 Lamia, Greece; 4 School of Mechanical and Aerospace Engineering, Nanyang Technological University, Singapore 639798, Singapore; 5 School of Sciences and Humanities, Nazarbayev University, Astana 010000, Kazakhstan; dongming.wei@nu.edu.kz; 6 Medical Center Hospital of the President’s Affairs Administration of the Republic of Kazakhstan, Astana 010000, Kazakhstan; madina_shapatova@mail.ru

**Keywords:** breast cancer, mammography, ultrasound, thermography, artificial intelligence, machine learning, deep learning

## Abstract

Breast cancer remains one of the most prevalent cancers worldwide, necessitating reliable, efficient, and precise diagnostic methods. Meanwhile, the rapid development of artificial intelligence (AI) presents significant opportunities for integration into various fields, including healthcare, by enabling the processing of medical data and the early detection of cancer. This review examines the major medical imaging techniques used for breast cancer detection, specifically mammography, ultrasound, and thermography, and identifies widely used publicly available datasets in this domain. It also surveys traditional machine learning and deep learning approaches commonly applied to the analysis of mammographic, ultrasound, and thermographic images, discussing key studies in the field and evaluating the potential of different AI techniques for breast cancer detection. Furthermore, the review highlights the development and integration of explainable artificial intelligence (XAI) to enhance transparency and trust in medical imaging-based diagnoses. Finally, it considers potential future directions, including the application of large language models (LLMs) and multimodal LLMs in breast cancer diagnosis, emphasizing recent research aimed at advancing the precision, accessibility, and reliability of diagnostic systems.

## 1. Introduction

Artificial intelligence (AI) could play a meaningful role in medical research and processing medical data. Modern AI tools can cover such tasks as prognosing disease risk, diagnosing different types of illnesses, analyzing treatment progress, and assessing outcomes of applied treatment. With the development of AI technologies, there is a good opportunity to process, at the same time, different types of information, such as medical tests, medical images, historical records, treatment tactics, and any follow-up information created during patient treatment. Advanced research in AI areas supports more accurate and personalized identification of the disease at the early stages, which enhances chances for successful treatment. Therefore, while there are many applications of AI in medicine, on the other hand, there is still room for AI development in an understandable, efficient, and open way.

The study discusses the main medical imaging models used to diagnose breast cancer, such as mammography, ultrasound, and thermography. Along with this the review covers AI methods such as traditional machine learning (ML) and deep learning (DL) algorithms for mammography, thermography, and ultrasound images processing. Many existing reviews focus on individual imaging modalities or AI techniques in isolation, leaving a gap in understanding how these approaches compare and complement one another across modalities. Furthermore, issues of explainability, data availability, and generalizability remain underexplored in the context of breast cancer imaging.

The aim of this review is therefore to provide a comprehensive comparison of AI applications in mammography, ultrasound, and thermography for breast cancer. Since the topic is interdisciplinary, this review has been designed to be useful and readable not only by AI experts or physicians but by the broader scientific community. We describe traditional ML and DL methods and performance metrics, and discuss the role of explainable artificial intelligence (XAI) and emerging LLM-based approaches. Finally, we highlight current challenges and future directions about research and clinical implementation in this rapidly evolving field.

The review distinguishes itself from existing surveys by offering a multimodal perspective on AI applications in breast cancer detection across mammography, ultrasound, and thermography—an approach that is rarely consolidated in the current literature. In addition to critically synthesizing recent advancements in machine learning and deep learning from 2020 to 2025, the paper uniquely incorporates emerging themes such as explainable AI (XAI), large language models (LLMs), and multimodal LLMs (MLLMs), which are only beginning to appear in the academic discourse.

The paper is structured as follows. The first section is the introduction. The second section briefly describes the main medical imaging methods used to detect breast cancer, such as mammography, ultrasound, and thermography, and presents relevant publicly available datasets. ML and DL techniques are described in the third section. The fourth section discusses metrics used to evaluate models and states the performance evaluation of ML and DL models. The fifth section presents the methodology of the study. The sixth section generally analyzes ML and DL, which are currently used in breast cancer detection in different research studies. The seventh section provides a further detailed analysis of AI techniques for mammogram, ultrasound images, and thermogram processing. The eighth section presents a discussion of breast cancer research studies. In addition, the paper discusses explainability for medical diagnosis and medical image recognition. Finally, the article discusses recent works using ML, DL, LLMs, and MLLMs for breast cancer detection.

## 2. Medical Image Analysis

To diagnose any abnormalities in the body, there are different image modalities, among which MRI, CT, PET, mammography, ultrasound, and duplex ultrasound could be highlighted. In this section, medical imaging techniques such as mammography, ultrasound, and thermography will be considered. These images are very helpful for diagnosing diseases, finding abnormal tissue, planning patient treatment, and understanding different health conditions [1]. In breast cancer diagnosis, the most common imaging modalities are mammography and ultrasound, with thermography serving as a supplementary method. These types of medical images are frequently used in AI-based research, largely due to the availability of established databases containing mammograms, ultrasound images, and thermograms.

### 2.1. Mammography Images

According to NCCN and WHO recommendations, mammography is widely considered the standard method for screening for women above age 40 [2,3]. Moreover, mammography is recommended for breast cancer screening by health organizations such as the American College of Radiology, Society of Breast Imaging (SBI), American Cancer Society, U.S. Preventive Services Task Force (USPSTF), National Comprehensive Cancer Network, European Society for Medical Oncology (ESMO), and others [4,5,6,7,8,9].

Mammography is based on low-dose X-rays that take detailed pictures of the breast, and show in detail soft and dense tissues, chest muscles, and fibro-glandular areas. Any abnormalities identified using a mammogram may require additional tests, such as biopsies, in order to determine the type of tumor: benign or malignant [2,3]. Mammography is mostly recommended for women above 40 as screening mammography, and for women under 40 as diagnostic mammography. Due to the anatomical characteristics of the breast in women of the specified age group.

The main advantage of screening mammography is the detection of tumors at an earlier stage; thereby, it allows for less extensive surgery, lower morbidity, and less aggressive adjuvant treatment. Since mammography is used in many countries, standardized instructions and descriptions of mammograms are well established. Through the years, mammography has gone through continuous improvement, and now there is digital mammography, 3D mammography/tomosynthesis, and contrast-enhanced mammography, which have improved sensitivity and specificity, especially in dense breasts. Integrating both 2D and 3D mammography yields higher diagnostic accuracy than relying on a single modality [10,11,12,13].

The main limitations of mammography are the high cost of the test, the uncomfortable procedure, and the use of radiation. In addition, mammography yields overdiagnosis, as benign changes or clinically insignificant tumors may be interpreted as cancer, leading to unwarranted medical procedures and treatment [14]. Lastly, due to anatomical features, the glandular and fibrous tissues in dense breasts appear white, like tumors, on mammograms. This can lead to false positive results and lead to unnecessary procedures and treatment [15]. Alternative mammographic techniques, including full-field digital mammography and digital breast tomosynthesis, may address some of the limitations of conventional mammography, particularly in women with dense breast tissue. Thus, mammography represents one of the most extensively investigated and debated diagnostic methods in medicine, while also being the most prescribed imaging procedure.

Over the years, many mammograms were collected by research doctors and stored in databases. According to the systematic review of Laws et al. [16], a total of 254 datasets were identified, of which 22 were openly accessible and 6 were available through managed access. The remaining datasets could not be accessed directly.

Listed below are some of the publicly available mammogram datasets most often referenced in research [1,17,18,19,20,21,22,23,24,25,26,27,28,29,30,31,32,33,34,35]:

- Digital Database for Screening Mammography (DDSM): This dataset includes 2620 mammograms that were originally on film and later scanned and divided into 43 volumes.
- Curated Breast Imaging Subset of the DDSM (CBIS-DDSM): This is an improved version of DDSM with bounding boxes, better mass segmentation, and decompressed images. It contains 10,239 mammograms, each with corresponding mask images.
- INBreast: This contains 410 images from 115 patients. In 90 cases, both breasts had cancer. It includes breast mass, calcifications, asymmetries, and distortions.
- Mini-MIAS: A dataset with 322 mammogram images and ground truth markers indicating possible abnormalities.
- BCDR (Breast Cancer Digital Repository):
  ○ BCDR-FM: Film-based mammography database.
  ○ BCDR-DM: Full-field digital mammography database.
  ○ Both include normal and abnormal cases, along with clinical details.
  ○ The BCDR-FM has 1010 cases (998 women, 12 men), 104 lesions, and 3703 mammograms taken from 1125 studies in Mediolateral Oblique (MLO) and Craniocaudal (CC) views.

Table 1 contains some of the mammogram databases commonly used in the research studies. These datasets provide valuable resources for breast cancer detection, diagnosis, and for conducting AI-based research in medical imaging.

### 2.2. Ultrasound Images

Ultrasound imaging is an alternative tool for breast cancer screening, especially applicable for women with dense breast tissue. It relies on high-frequency sound waves, instead of using ionizing radiation to create images.

Among the key advantages of ultrasound are its non-invasive, cost-effective, and safer choice for younger women, who may require several screenings. In young, dense breasts, ultrasound can clearly differentiate fluid-filled cysts, solid masses, and normal breast tissue [36,37]. Moreover, studies demonstrate that incorporating ultrasonography as a complement to mammography increases detection accuracy in women with dense breast tissue. According to [38], ultrasound raised cancer detection by 1.9–3.5 per 1000 women with dense breasts under the age of 50.

Despite its obvious advantages, ultrasound has a restricted capability to identify calcifications. Compared to mammography, it has reduced specificity and depends on the experience of an ultrasound doctor to ensure accurate testing [39,40].

Over the years, a large number of ultrasound images have been collected and stored in databases, which are useful for AI-based research. There are several available datasets useful for the research [41,42,43,44,45,46,47,48]:

- Breast ultrasound images (BUSI) dataset contains 780 grayscale ultrasound images of 600 female patients: normal–133 images, benign–437 images, and malignant–210 images.
- Open access series of breast ultrasonic data (OASBUD) contains 200 ultrasound scans of 78 women aged between 25 and 75 years: 100 breast lesions (52 malignant, 48 benign).
- UDIAT breast ultrasound dataset (UDIAT) contains 163 ultrasound images: benign–109 images and malignant–54 images.
- BrEaST ultrasound dataset contains 256 breast ultrasound scans from 256 patients, including 266 benign and malignant segmented lesions.
- QAMEBI ultrasound database contains 232 breast ultrasound images, including 123 benign and 109 malignant breast lesions.
- BUS-BRA breast ultrasound dataset contains 1875 anonymized images from 1064 female patients, divided into 722 benign and 342 malignant cases.

The most detailed compilation of ultrasound image databases is presented in Table 1.

### 2.3. Thermography Images

Thermography was first introduced in 1982. For medical issues, it was used later, but is still not approved as a medical tool [49,50,51]. Thermography could be used as an adjunct tool for various medical problems, including breast cancer. In order to capture temperature differences and make a diagnosis, thermography uses thermal infrared cameras. The differences in the temperature distribution on the surface of the breast may indicate the presence of a tumor inside the breast [51,52,53].

The resulting thermograms show temperature patterns, helping in the early identification of abnormalities. Thermography could be used as an additional tool for first entry screening, in order to control any abnormal temperature distribution on the surface of the breast. In case of abnormal temperature distribution on the surface of the breast, a patient could be redirected to deeper screening using mammography or MRI.

The advantages of thermography include reduced reliance on X-rays (as used in the case of mammography), lower overall cost when compared to mammography and MRI, and greater accessibility owing to its affordable and portable equipment, which is especially valuable in resource-limited settings.

One of the main limitations of thermography is that it only captures surface-level measurements, thereby excluding the diagnosis of tumors located deeper within the tissue [52,53]. In addition, portions of the image are generated through extrapolation of data, making the process susceptible to inaccuracies that could result in diagnostic errors.

Due to the lower popularity of thermography compared with mammography or ultrasound, only a limited number of thermograms are available in databases. One of the most popular thermogram databases is the Mastology Research Database. It is widely used in thermal imaging research and includes thermograms from 287 individuals, aged 23 to 120 years. Table 1 contains the complete list of existing databases.

### 2.4. Publicly Available Datasets

Table 1 presents a summary of the most frequently used databases in breast cancer classification studies, including, but not limited to:

- Breast Cancer Data Repository (BCDR)
- Digital Database for Screening Mammography (DDSM)
- INBreast
- Mammographic Image Analysis Society (MIAS)/Mini-MIAS
- Wisconsin Breast Cancer Dataset (WBCD)
- Wisconsin Diagnosis Breast Cancer (WDBC)
- Image Retrieval in Medical Applications (IRMA)
- Breast Cancer Histopathological Image (BreakHis)
- Breast Ultrasound Images Dataset (BUSI)
- Open Access Series of Breast Ultrasonic Data (OASBUD)
- UDIAT Breast Ultrasound Dataset (UDIAT)
- BrEaST Ultrasound Dataset
- The Mastology Research with Infrared Image (DMR-IR)
- NIRAMAI Health Analytix

Presented datasets are frequently used for breast cancer detection, diagnosis, and AI-based classification research.

According to research studies conducted recently, medical image analysis has seen significant progress with the use of AI, specifically ML and DL. By applying image processing and ML techniques, doctors can now detect and diagnose cancer earlier, improving the accuracy of breast cancer diagnosis.

## 3. Machine Learning and Deep Learning Techniques

### 3.1. Traditional Machine Learning and Deep Learning

Machine learning (ML) is a branch of AI that refers to a collection of methods that analyze data to identify patterns. As a result, it can then be applied to forecast future data or predict various outcomes of interest [1].

Deep learning (DL) is a specialized field within ML and AI. According to [1,54], DL can analyze medical images and provide useful information about disease prognosis, treatment responsiveness, and any other related characteristics regarding one or another disease. Among other tools, the most common techniques are convolutional neural networks (CNNs), Boltzmann machines, and deep neural networks (DNN).

**Figure 1:**
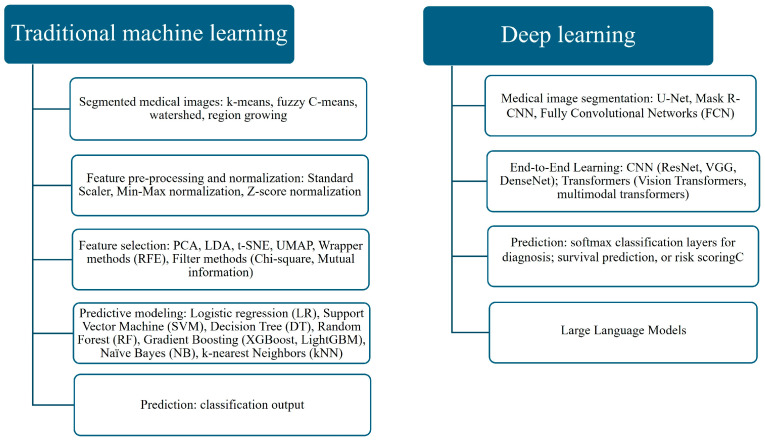
Typical architecture and workflow of AI systems for predictive modeling.

There are two main architectures and typical workflows used to create AI applications for medical imaging (Figure 1):

- Traditional ML, which usually uses manually selected features known as radiomic features [54,55] extracted from segmented images.
- The DL approach, which involves deep feature extraction or end-to-end learning directly from images.

Figure 1 demonstrates the differences between the two approaches of traditional ML and DL. It emphasizes the difference between the workflows and approaches of processing medical images. The workflow in traditional ML is manual and segmented. It requires preprocessing and normalization in order to extract features from the image. To execute preprocessing, algorithms such as k-means clustering, fuzzy c-means, or watershed methods are used to isolate regions of interest from mammography, ultrasound, or thermography images. Further, the extracted features are manually selected, and later used for training modes, as a result trained model generates predictions and classification. Feature preprocessing and normalization techniques, including standard scaling, Z-score normalization, or min–max scaling, are applied to ensure data consistency. At the feature selection stage, dimensionality reduction and filtering methods like PCA, LDA, t-SNE, UMAP, recursive feature elimination, or chi-square tests are employed to retain the most relevant information. Predictive modeling is carried out using algorithms such as logistic regression (LR), SVM, decision trees (DT), random forests (RF), gradient boosting methods (XGBoost, LightGBM), naïve Bayes, or k-Nearest Neighbors (kNN). Finally, these models generate predictions, showing the possibility of the presence of a tumor in the breast.

On the other hand, DL models are more automated, and they exclude the need for manual segmentation of the image. The created network automatically learns feature extraction and selection during training, and in the end, produces predictions. Specialized architectures like U-Net, Mask R-CNN, or fully convolutional networks (FCNs) are widely applied to medical image segmentation. For feature learning and classification, convolutional neural networks (CNNs) dominate. For temporal ultrasound data, Recurrent Neural Networks (RNNs) and LSTMs are used. Transformer models are increasingly adopted for multimodal integration. These models ultimately produce predictions such as diagnostic classifications and risk scores. One of the main advantages of DL models is optimizing network performance for specific tasks. Such models establish sophisticated connections within huge datasets, as well as in small datasets, and still provide reliable results. Therefore, it is widely employed in medical imaging. Hence, Figure 1 emphasizes how DL automates and integrates multiple steps of traditional ML into a streamlined, end-to-end process [56,57,58,59,60,61,62,63].

Hybrid or ensemble models combine the strengths of both ML and DL. They are often employed by stacking, bagging, boosting, or fusing data from multiple imaging modalities. Furthermore, another case of hybrid models is the XAI tools, including Grad-CAM, LIME, SHAP, and attention mechanisms, which are increasingly incorporated to improve transparency. Advanced transparency is beneficial for doctors and physiologists, as it assists in interpreting the reasoning behind predictions.

In spite of the different approaches and workflows of ML and DL methods, they both explore supervision and training principles. Both of the models are able to combine additional data as extra parameters, such as demographic information, risk factors, and molecular profiles, to enhance the predictive accuracy of the models. In particular, multimodal imaging [56,57,58,59] and multiomics data [57,58,59] can be utilized to gain better medical decision making concerning the analyzed region. At the same time, combining different types of data into a single model is a key challenge for both ML and DL models. One more challenge of training ML and DL models is the quality of data collection and its processing. The principle of “garbage in, garbage out” is especially relevant in data collection for AI-driven research. The accuracy and reliability of the results entirely depend on the quality of the input images. In order to collect a large dataset, frequently, medical images are collected from multiple centers. This leads to variations in equipment, imaging techniques, and clinical protocols, which may contribute to data inconsistency and complexity. Therefore, in order to obtain accurate and reliable results, the quality of data is one of the important parts of the data-driven AI research [61,62,63].

### 3.2. Large Language Models

Large language models (LLMs) are a type of language model that uses the transformer architecture, trained on massive datasets with billions of parameters. The development of large language models (LLMs) such as GPT-4, LLaMA, Gemini, Grok, and DeepSeek demonstrates a transformative shift in the field of medical AI, particularly in breast cancer (BC) diagnostics. Based on transformer architectures and being pre-trained on massive text corpora, these models have the ability to interpret sophisticated language, analyze multimodal clinical data, and generate semantically coherent responses. The LLMs’ capacity to process clinical narratives, extract structured information, and generate personalized clinical insights leads to a new era of precision oncology.

The key benefit of these models is their applicability across the entire breast cancer (BC) diagnostic process. For instance, models such as ChatGPT-4 and Gemini have shown the ability to align with recommendations of multidisciplinary tumor boards, create treatment strategies, and provide decision support with reported accuracies ranging from 70–98% [64]. The integration of the models into medical practice demonstrates potential in a number of issues. Firstly, LLMs can flag signs of malignancy more consistently than traditional rule-based systems due to early detection through the synthesis of data from imaging reports (mammograms, ultrasound, MRI). Secondly, the integration of genetic and biomarker data (e.g., BRCA1/2 mutations) in order to assess patient-specific risk profiles through the stratification of risks by such tools as BioGPT or ClinicalBERT. Finally, a holistic understanding of disease progression can be provided by multimodal data interpretation, specifically models trained on both structured (e.g., lab results) and unstructured (e.g., physician notes) data.

Furthermore, ChatGPT has shown effectiveness in translating sophisticated oncology concepts into language that is understandable to non-specialists. This improves patients’ understanding of treatment pathways as well as side effects and required changes in lifestyles [64]. Additionally, LLMs improve shared decision making by allowing patients to request treatment or surgical procedure options. It may also reduce anxiety and promote treatment adherence.

According to [53,65,66,67], limitations remain that hinder the immediate clinical translation of LLMs for breast cancer, even though their potential is substantial. The studies highlight the scarcity and diversity of data, while LLMs in medicine require rich, diverse, and high-quality data in order to be robust. Current models are reduced in terms of generalizability across diverse populations because of limited datasets. Secondly, there is the risk of hallucinating or misrepresenting clinical facts by the LLMs, that, unlike in supervised learning, can occur. The lack of ability to interpret and explain the obtained results undermines the trust in high-stakes clinical environments. Third is the lack of benchmarks and standards, which means that the field lacks standardized assessment protocols. This causes difficulties in comparing performance across various models and clinical contexts. Finally, given that LLMs work with confidential data of patients, the security and ethical issues are subject to risks of cybersecurity, the spread of bias, and issues related to informed consent and data management.

### 3.3. Multimodal Large Language Models

Multimodal large language models (MLLMs) are considered advanced AI models that process and generate numerous types of data, including text, images, audio, or other modalities, within a single framework. Compared to traditional LLMs, multimodal LLMs integrate and analyze these diverse inputs to perform tasks such as creating image captions, visually answering questions, or generating text from images.

Multimodal LLMs usually combine such components as transformers (for text) with vision models (e.g., CNNs or ViTs) or audio processing modules, frequently using a shared embedding space to accommodate different modalities. MLLMs leverage DL to handle sophisticated multi-data-type tasks. For instance, models such as GPT-4o (OpenAI), LLaVA, CLIP-ViT, or Flamingo can process text and images, while other models like Whisper (OpenAI) handle audio and text. These models can execute such tasks as generating image descriptions, answering questions about visual content, or even creating images from text prompts (e.g., with diffusion models integrated).

MLLMs are trained on various datasets merging text, images, or audio, frequently requiring big data to align modalities effectively. Hence, these models are used in such areas as autonomous systems, content generation, medical imaging analysis, and interactive assistants.

### 3.4. Explainable AI for Medical Diagnosis and Medical Image Recognition

Explainable AI (XAI) refers to methods and techniques in AI that make the results of AI systems understandable and interpretable by humans and future strong AI systems.

Explainable AI is important since users and stakeholders need to understand how and why an AI system makes a particular decision. This is a common practice and requirement in everyday human decision making. Human experts always explain the reasons behind their decisions in almost all domains. In regulated activities like finance, healthcare, and law, it is necessary to explain decisions for scientific, legal, and ethical reasons. Imagine a situation where different AI systems give different diagnoses. This is a realistic situation that can happen in the case of nontrivial problems. Just as human experts explain their reasoning before reaching a well-considered decision, AI systems should also be capable of providing explanations for how they arrive at their conclusions. XAI also helps to identify if a model is making decisions based on biased or unfair patterns in the data. Finally, explainability can guide debugging and improvement of AI systems since developers need explanations to improve the model’s performance.

It is obvious that medical diagnosis is not a trivial kind of decision-making process. It requires severe quality standards to be met since human lives can be at risk. It is well known that in several types of medical decisions, like diagnoses, experienced and specialized physicians may suggest different outputs. The same can happen among AI systems or among AI and human experts. The only solution to this problem is for AI systems to be able to provide explanations, like humans.

There are several different approaches towards XAI. The most notable and relevant for images include:

(1) LIME (local interpretable model) approximates the complex model locally around a prediction using a simple interpretable model (like a linear model). It perturbs the input data and observes changes in prediction to estimate feature importance [68].
(2) SHAP (SHapley Additive exPlanations) is based on cooperative game theory (Shapley values). It measures each feature’s contribution by calculating its marginal impact across all possible feature combinations [69].
(3) Saliency maps/gradient-based methods use gradients of the output with respect to input features (e.g., pixels) to determine which features most influence the prediction. Most relevant in image classification (e.g., CNNs) [70,71].
(4) Feature importance scores methods like tree-based models (e.g., random forests, XGBoost) provide built-in feature importances. It is possible to include permutation importance (shuffling a feature and measuring performance drop) [72,73].
(5) Counterfactual explanations find the smallest change to the input that would result in a different prediction [74].
(6) Anchors identify a set of rules (“anchors”) that “lock in” a prediction. If these rules are met, the model’s prediction is likely to stay the same [75].
(7) Surrogate models train a simpler, interpretable model (like a decision tree) to mimic the predictions of a more complex model [76].
(8) Models using knowledge representation schemes. It is possible to use one of the previous methods to identify the critical parts (for diagnosis) of a medical image and subsequently to calculate various characteristic quantities of these subsets of the image. Then, a Bayesian Network (BN) can be constructed with informational nodes representing those quantities. The causal structure of the BN reveals immediately the most influential factors for the classification and how these factors interact and influence each other [77,78,79,80].

For medical imaging, the most relevant and widely used explainable AI (XAI) methods are visualization-based and gradient-based attribution methods, because they align with how radiologists and medical professionals interpret images (e.g., highlighting regions of interest). The most relevant for medical images are approaches 1, 2, 3, 4, and 8, although 5, 6, and 7 can be used in hybrid systems that combine different approaches.

### 3.5. What Clinicians Should Know

Tools such as gradient-weighted class activation mapping (Grad-CAM) and layer-wise relevance propagation (LRP) can help clinicians better understand AI decisions and potentially build trust in their use. In breast imaging, many XAI tools generate maps that highlight areas that are most influential for the model’s decision. These overlays resemble how radiologists interpret suspicious regions and can be used as a secondary reference. However, they should not be taken as absolute evidence, since explanation maps can vary depending on the model used. A major concern is saliency map instability, where the same input image may generate different explanation maps depending on the model architecture or training conditions. This variability underscores that XAI outputs should be regarded as supportive evidence rather than definitive diagnostic proof.

## 4. CAD Systems Performance Evaluation

There are four main metrics to classify breast cancer diagnoses. For correct breast cancer diagnoses, the doctors use either true positive (*TP*) or true negative (*TN*), while an incorrect diagnosis falls under false positive (*FP*) or false negative (*FN*) metrics.

There are six commonly used metrics to characterize the accuracy of model classification, including accuracy, sensitivity, precision, F-measure, area under the curve (*AUC*), and volume under the receiver operating characteristic (*ROC*) surface [81]. Below is the brief explanation of each metric.

Accuracy (*ACC*) evaluates the proportion of correctly classified cases out of the total number of instances. It is determined using Equation (1).

(1) $$ACC=\frac{TP+TN}{TP+TN+FP+FN}$$

Sensitivity (*Sn*), or recall, indicates the proportion of actual positive cases that are correctly identified. In other words, it measures how accurately the system detects patients with breast cancer. It is calculated using Equation (2).

(2) $$Sn=\frac{TP}{TP+FN}$$

Specificity (*Sp*) measures the accuracy of negative predictions, indicating the proportion of correctly identified non-cancerous cases. It represents how well the system distinguishes healthy individuals from unhealthy. Specificity is calculated using Equation (3).

(3) $$Sp=\frac{TN}{\left(TN+FP\right)}$$

Precision (*Pr*) indicates the proportion of predicted positive cases that are actually correct, specifically measuring the accuracy of identifying breast cancer cases. In medical imaging diagnosis, both sensitivity (*Sn*) and precision (*Pr*) should be high to minimize misdiagnosis. It is defined using Equation (4).

(4) $$\mathrm{Pr}=\frac{TP}{TP+FP}$$

F-measure or *F_β_* score balances both sensitivity, also named recall, and precision by using their harmonic mean, ensuring a more reliable assessment by penalizing extreme values. It can be calculated using Equation (5).

(5) $$F_{\beta }=\frac{(1+\beta^{2})\times Presicion\times Recall}{(\beta^{2}\times Presicion)+Recall}$$

*β* = 1: This is the standard *F1*-score, which gives equal weight to precision and recall.

*β* > 1: Favors recall (sensitivity), making it more useful for minimizing false negatives.

0 < *β* < 1: Favors precision, making it more useful for minimizing false positives.

*AUC* (area under the curve) indicates how well the model performs under various conditions. A higher *AUC* value indicates better model performance. It can be determined using Equation (6) or Equation (7). Equation (6) is a version of *AUC* that tells how well a classifier ranks positive instances above negative ones. If all positive instances are ranked above all negative instances, *AUC* = 1; if the ranking is random, then *AUC* ≈ 0.5. Equation (7) is another standard definition of *AUC*, and it is probabilistic and pairwise in nature. It computes the proportion of all positive–negative pairs where the classifier gives a higher score to a positive instance than to a negative one. If all positive scores are higher than all negatives, then *AUC* = 1; if scores are random, then *AUC* ≈ 0.5; if the model ranks negatives above positives, then *AUC* < 0.5.

(6) $$AUC=\frac{\sum R_{i}\left(I_{p}\right)-I_{p}(I_{p}+1)/2}{I_{p}+I_{n}}$$

where $I_{p}$ and $I_{n}$—number of positive and negative images of breast cancer.

*R_i_* is the rating of the *i*-th positive image.

Finally, this definition gives:

(7) $$AUC=\frac{1}{\left|P\right|\cdot \left|N\right|}\sum_{x_{p}\in P}\sum_{x_{n}\in N}II(s(x_{p})>s(x_{n}))$$

*s*(*x*)—predicted score/probability for sample *x*

*II* is 1 if the positive image gets a higher score than the negative; otherwise, it is 0.

Recently, a quality score for imaging-based AI models [58,59] has been announced to evaluate their consistency by assessing numerous phases in the examination workflow. Even though its efficiency is still deliberated, it provides a convenient means for inventors and scholars to confirm that all compulsory features and validation assessments are encompassed in AI model progress.

## 5. Literature Search Methodology

A systematic search was conducted to identify relevant studies on AI applications in breast cancer detection using mammography, ultrasound, and thermography. Scopus, Web of Science, and Science Direct databases were reviewed. The search terms included combinations of the following:

- “breast cancer” AND (“mammography” OR “ultrasound” OR “thermography”);
- “artificial intelligence” OR “machine learning” OR “deep learning”;
- “explainable AI” OR “XAI”;
- “large language models” OR “LLMs” OR “multimodal AI”;

The search was restricted to peer-reviewed articles in English published between 2020 and 2025. Studies were included if they reported on the use of ML, DL, or XAI techniques for breast cancer detection, classification, or diagnosis in at least one of the three imaging modalities. Exclusion criteria were studies not related to breast imaging, those without sufficient methodological detail, or purely technical papers without clinical application.

Given the enormous number of publications on AI and breast cancer detection, we applied strict inclusion criteria focusing on studies with clearly described AI methods and evaluation metrics; research demonstrating direct relevance to clinical or diagnostic applications; papers providing sufficient methodological detail for reproducibility; reference lists of key articles and reviews were also screened to identify additional relevant publications.

This approach was intended to ensure the quality and clinical relevance of the included studies rather than to provide an exhaustive list. As a result, for some modalities—such as ultrasound—fewer studies met all inclusion criteria, which explains why only a limited number are presented despite the broader literature.

## 6. Breast Cancer Detection and Classification Using Machine Learning and Deep Learning

**Figure 2:**
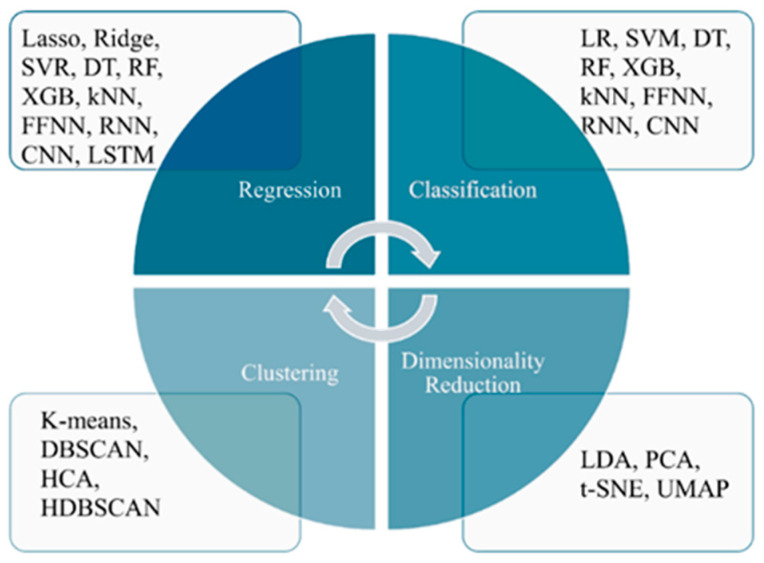
List of machine learning algorithms used for different types of applications.

Different algorithms for medical image processing and modeling are required with the development of ML tools and the diversity of data collection. A variety of specialized algorithms were developed and used for different tasks of ML, such as regression, classification, clustering, and dimensionality reduction. ML algorithms that can be used for the abovementioned tasks are demonstrated in Figure 2. Below is a brief discussion of the available ML algorithms for each task.

Regression implicates foreseeing continuous numeric outcomes, comprising such approaches as: Lasso regression, ridge regression, support vector regression (SVR), decision trees (DT), random forests (RF), XGBoost (XGB), k-Nearest Neighbors (kNN), feedforward neural networks (FFNN), recurrent neural networks (RNN), convolutional neural networks (CNN), and long short-term memory networks (LSTM) (Figure 2).

Classification predicts categorical outcomes or class labels and includes such methods as logistic regression (LR), support vector machines (SVM), decision trees (DT), random forests (RF), XGBoost (XGB), k-Nearest Neighbors (kNN), feedforward neural networks (FFNN), recurrent neural networks (RNN), and convolutional neural networks (CNN) (Figure 2).

Clustering involves grouping data points based on similarity, with no predefined labels. The method includes k-means, DBSCAN, hierarchical cluster analysis (HCA), and hierarchical density-based spatial clustering (HDBSCAN) (Figure 2).

Dimensionality reduction techniques reduce the complexity of the data, preserving essential features, and include the following methods: linear discriminant analysis (LDA), principal component analysis (PCA), t-distributed stochastic neighbor embedding (t-SNE), and uniform manifold approximation and projection (UMAP) (Figure 2).

**Figure 3:**
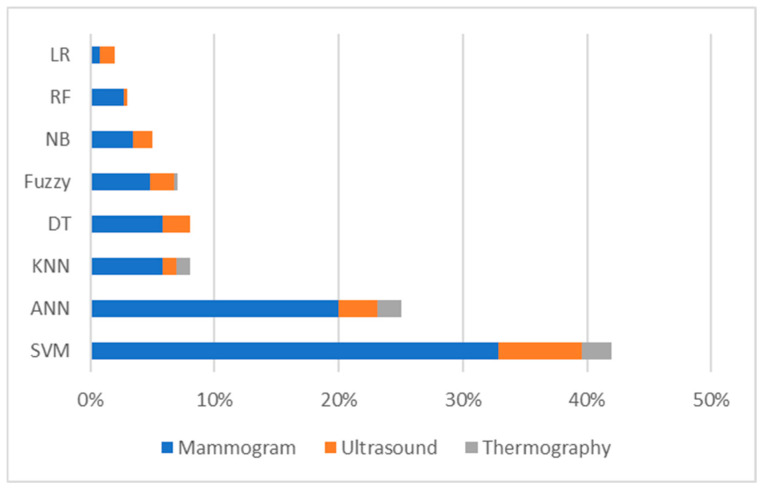
Machine learning algorithms applied to breast cancer detection using mammogram, ultrasound, and thermography.

Continuous development and innovations in AI facilitate conducting more precise, scalable, and insightful research across different fields, including healthcare, specifically medical imaging. Figure 3 shows the distribution of published studies that used different ML methods for different tasks, exploring mammography, ultrasound, and thermography modalities. The figure is based on the information obtained from Scopus, Web of Science, and ScienceDirect databases.

The analysis of the abovementioned databases demonstrates that approximately 44% of the studies published between 2020 and 2025 employed support vector machine (SVM) as a classifier for breast cancer detection. SVM was most frequently associated with mammogram images, although some studies also utilized ultrasound and, to a lesser extent, thermography images. The second most used method was ANNs, including RNNs, CNNs, and other transformer-based neural networks, which also highlights a strong focus on mammographic data, with fewer applications involving ultrasound and thermography images (Figure 3).

Other approaches, such as kNN, DT, fuzzy logic, naïve Bayes (NB), RF, and LR, contributed between 2% and 10% of searched studies. These methods also primarily relied on mammogram features, with smaller contributions from ultrasound and minimal use of thermography images.

Among all methods, LR showed the lowest overall contribution, with only slight application across the three imaging modalities. This distribution is clearly illustrated in Figure 3, which shows how published studies are spread across different diagnostic techniques, including mammography, ultrasound, and thermography modalities.

Recently, LLMs have gained growing interest as another advanced AI model. These models are based on transformed architectures and consist of those pre-trained on large-scale text datasets [82]. The use of the transformer-based model is explained by significant improvement of the model performance, especially with increases in model size, the volume of training data, and available computational resources. Examples of such models include ChatGPT (developed by OpenAI), DeepSeek (by DeepSeek AI), LLaMA (by Meta), and Grok (by xAI) [83,84].

In terms of breast cancer, LLMs can be used with the aim of improving the accuracy and effectiveness of the workflow. In the near future, such models can be explored to integrate different clinical data, such as text, various images, genomic information, etc., by such to manage a large amount of information in a short time, automatically, and create comprehensive decisions about illness [85]. For patients, LLMs can lead to having personal doctors in their pocket, which can interpret various medical images, produce user-friendly explanations and conclusions, thereby assisting patients to better understand their disease [85,86]. The studies conducted in the field of LLMs are closely considered in Section 7.4 of the current review.

## 7. Artificial Intelligence for Breast Cancer Detection: Machine Learning, Deep Learning, and Hybrid Algorithms

In recent years, ML and DL, and hybrid algorithms have been developed to extract specific features and enhance the effectiveness of medical image analysis. This section presents an overview of ML and DL, and hybrid approaches, used for breast cancer detection and classification across three medical imaging modalities: mammography, ultrasound, and thermography (Table 2, Table 3 and Table 4).

### 7.1. Machine Learning, Deep Learning, and Hybrid Algorithms for Breast Cancer Detection Using Mammograms

Numerous research studies have explored the use of mammography images for detecting and classifying breast cancer by employing ML, DL, or hybrid algorithms. Table 2 presents some publications that apply ML, DL, and hybrid techniques in analyses of mammograms.

**Table 2:** Studies used ML, DL, or hybrid algorithms for breast cancer detection using mammograms.

Reference	ML, DL, Hybrid Algorithms	Dataset	Performance Evaluation
Sha, Z.; Hu, L.; Rouyendegh, B.D. (2020) [87]	Hybrid: CNN, SVM	MIAS, DDSM	*Sn* is 96%, *Sp* is 93%, *PPV* is 85%, *NPV* is 97%, *ACC* is 92%.
Sapate, S.; Talbar, S.; Mahajan, A.; Sable, N.; Desai, S.; Thakur, M. (2020) [88]	ML: fuzzy c-means algorithm; SVM;	Tata Memorial Centre (TMC), Mumbai, India; BIRADS	*Sn* is 75.91% at 0.69 FPs/I and *Sn* is 73.65% at 0.72 FPs/I.
Mansour, S.; Kamal, R.; Hussein, S.A.; Emara, M.; Kassab, Y.; Taha, S.N.; Gomaa, M.M. (2025) [89]	AI	Internal dataset	*Sn* is 73.4%, *Sp* is 89%, *ACC* is 78.4%.
Malherbe, K. (2025) [90]	AI Breast	Internal dataset: Daspoort PoliClinic in Gauteng, South Africa	97.04% were negative, 2.46% were positive, and a single patient was classified with a BIRADS2 score.
Hernström, V.; Josefsson, V.; Sartor, H.; Schmidt, D.; Larsson, A.-M.; Hofvind, S.; Andersson, I.; Rosso, A.; Hagberg, O.; Lång, K. (2025) [91]	AI system	Swedish national screening program, women recruited at four screening sites in southwest Sweden (Malmö, Lund, Landskrona, and Trelleborg)	AI-supported screening led to a 29% increase in cancer detection.
Nour, A.; Boufama, B. (2025) [92]	CNN: U-Net deep learning model; ACM	Chinese Mammography Database (CMMD)	*ACC* is 0.9734; validation Loss is 0.037; average Dice Coefficient is 0.813; average intersection over Union is 0.891.
Umamaheswari, T.; Babu, Y.M.M. (2024) [93]	Hybrid DL model combining CNN (EfficientNetB7) and Transformer (ViT): ViT-MAENB7 model	The Complete Mini-DDSM Dataset;	*ACC* is 96.6%; recall is 96.6%; *Sp* is 96.6%; *Pr* is 93.4%; *F1*-score is 94.9%.
Mannarsamy, V.; Mahalingam, P.; Kalivarathan, T.; Amutha, K.; Paulraj, R.K.; Ramasamy, S. (2025) [94]	SIFT-CNN; Fuzzy-based decision tree	CBIS-DDSM dataset	For normal cases: *ACC* is 98.98%; *Sp* is 96.28%; *Sn* is 94.78%. For benign cases: *ACC* is 99.74%; *Sp* is 95.76%; *Sn* is 93.64%. For malignant cases: *ACC* is 98.89%; *Sp* is 93.59%; *Sn* is 95.82%.
Puttegowda, K.; Veeraprathap V; Kumar, H.S.R.; Sudheesh, K.V.; Prabhavathi, K.; Vinayakumar, R.; Tabianan, K. (2025) [95]	ML: Faster R-CNN; YOLOv3; RetinaNet	DDSM, INbreast, AIIMS	For normal cases: recall is 99%, *Sp* is 98.79%, *Pr* is 98.59%, *F1*-score is 98.95%, *AUC* is 99.58%. For benign cases: recall is 93.56%, *Sp* is 98.57%, *Pr* is 96.38%, *F1*-score is 94.67%, *AUC* is 97.59%. For malignant cases: recall is 92.78%, *Sp* is 97.43%, *Pr* is 94.71%, *F1*-score is 92.43%, *AUC* is 99.87%.
Ahmad, J.; Akram. S.; Jaffar. A.; Ali, Z.; Bhatti, S.M.; Ahmad, A. et al. (2024) [96]	CAD system: ML (SVM) & computer vision techniques	CBIS-DDSM	*ACC* is 99.16%, *Sn* is 97.13%, *Sp* is 99.30%.
Gudur, R.; Patil, N.; Thorat, S. (2024) [97]	Integration of CNN, ResNet50, RNN	RSNA	*ACC* is 97%; *AUC* is 0.68; *Pr* is 60%; recall is 80%; *F1*-score is 0.18.
Mahmood, T.; Saba, T.; Rehman, A.; Alamri, F.S. (2024) [98]	DCNN-based models: CNN + LSTM and CNN + SVM	MIAS, INbreast	*ACC* is 98%; *Pr* is 97%; recall is 97%; *Sn* is 97%; *F1*-score is 0.97.
Muduli, D.; Dash, R.; Majhi, B. (2020) [99]	LWT; MFO-ELM	MIAS, DDSM	*ACC* is 98.80%, *AUC* is 0.99.

Sha et al. (2020) [87] proposed a technique based on CNNs for the segmentation of apprehensive regions in mammographic images. During pre-processing, to reduce noise in the initial images, the median filter was explored. Further, to separate cancerous regions on the image CNN-based optimizer was applied. Feature extraction was conducted to reduce computational load and enhance diagnostic accuracy. The researchers’ advanced optimization approach aimed to identify the most suitable characteristics for the CNN, which can segment possible areas with tumors. Performance of the suggested system was compared with other modern tools to test the system; MIAS and DDSM mammographic databases were employed. The proposed approach achieved 96% sensitivity, 93% specificity, 85% positive predictive value (PPV), 97% negative predictive value (NPV), and 92% overall accuracy.

Mansour et al. (2025) [89] conducted research on estimating the efficiency of AI to diagnose breast cancer using digital mammograms. The study employed 1998 mammograms, produced from 2020 to 2023, to establish relations between AI-identified marked regions of interest and histologically inveterate missed cancers. AI effectively labeled 54% of earlier missed carcinomas and had a detection rate of 88% in current mammograms. The model achieved a sensitivity of 73.4%, specificity of 89%, and overall accuracy of 78.4%. The obtained results showed that AI systems improve the diagnosis of cancerous tumors, especially in young women, who usually have dense breast tissue. Thus, notable enhancements could be reached in the diagnosis of breast cancer at early stages in dense breast tissue.

The same conclusion, that AI could enhance breast cancer detection at the early stages, was made by the MASAI study [91]. This Sweden study involved over 105,000 women and focused on estimating the effect of AI-assisted mammography screening based on the Transpara system. Compared to the standard routine, the AI-supported system demonstrated a 29% growth in cancer diagnosis identification, with higher positive predictive values of recalls and lower false positive values. It was estimated that the workload of radiologists decreased by 44.2%. Thus, the results obtained supported the hypothesis that an AI-assisted system can significantly optimize the radiologist’s workload and screening workflow. Furthermore, improve efficiency and, more importantly, enhance breast cancer diagnosis.

A study by Nour et al. (2025) [92] introduced a hybrid algorithm that considers exploring DL and active contour modeling. The aim of the hybrid algorithm is to analyze mammograms automatically. The methodology suggested in the study combines feature extraction capabilities of deep CNNs with the boundary delineation accessible through active contour models (ACMs). Performance metrics showed an accuracy of 97.34%, a Dice coefficient of 0.813, and an Intersection over Union (IoU) of 0.891. These findings show the usefulness of the suggested model for lesion segmentation in mammograms, which leads to accurate diagnosis and decreases the number of false positive or false negative results. However, extra assessment on larger and diverse datasets is required to validate the model’s generalizability across diverse patient populations.

A study by Umamaheswari et al. (2024) [93] suggested a breast cancer diagnosis system that utilizes 3D mammogram images that were publicly available. The first step was segmentation, which was realized by using Adaptive Thresholding with Region Growing Fusion Model (AT-RGFM). Further, the segmentation process was optimized by exploring the Modified Garter Snake Optimization Algorithm (MGSOA). Finally, a tumor-defining process was utilized by the Vision Transformer-based Multiscale Adaptive EfficientNetB7 (ViT-MAENB7) model. This hybrid DL model leverages both transformer-based and convolutional architectures to perform multi-scale image analysis, enabling it to detect tumors with high precision across varying levels of detail. The model achieved an accuracy of 96.6%, with a recall of 96.6%, specificity of 96.6%, precision of 93.4%, and an F1-score of 94.9%. These results support the effectiveness of the hybrid algorithm for breast cancer diagnosis in mammograms.

A study by Mannarsamy et al. (2025) [94] presented the SIFT-CNN Integrated Fuzzy Decision Tree-based Breast Cancer Detection (SIFT-BCD) method for the early and efficient breast cancer diagnosis. The study utilized the CBIS-DDSM dataset. To remove noise and preserve edge information, the images are first processed using a trilateral filtering technique. The denoised images are then passed through a region of interest (ROI)-based U-Net model for accurate segmentation of relevant breast tissue areas. For feature extraction, a hybrid approach combining scale-invariant feature transform (SIFT) with CNN was used to capture both low-level and deep visual features. The extracted features are classified using a fuzzy decision tree, which categorizes images into three diagnostic groups: malignant, benign, and normal. The model’s performance showed three types of classification: normal cases, benign cases, and malignant cases. For normal cases, performance metrics showed accuracy of 98.98%, specificity of 96.28%, and sensitivity of 94.78%. For benign cases, performance metrics were accuracy of 99.74%, specificity of 95.76%, and sensitivity of 93.64%. For malignant cases, performance metrics were calculated as accuracy 98.89%, specificity 93.59%, and sensitivity 95.82%. However, the model’s reliance on the CBIS-DDSM dataset may limit its generalizability due to restricted patient diversity and imaging conditions.

A study by Puttegowda et al. (2025) [95] presented a multi-model framework utilizing DDSM, INbreast, and Indian AIIMS mammograms datasets, focusing on Indian women. The framework integrates R-CNN, RetinaNet, and YOLOv3 object detection models and suggests advancements as a photometric transform network and perturbation-based attention analysis for interpretability and performance. The system achieved high classification results: accuracy 98.8%, sensitivity 98.5%, and AUC 0.99 by using the YOLOv3 model.

Ahmad et al. (2024) [96] proposed a comprehensive CAD system based on DL for mammogram classification and breast cancer detection. The system consists of the YOLO-V7 model for lesion recognition, the Associated-ResUNet for segmentation, and BreastNet-SVM (AlexNet-based) for classification. In addition, the study highlighted that fused model strategies and multi-resolution input were used for model performance optimization. The study was performed utilizing the CBIS-DDSM dataset. The system achieved an accuracy of 98.5%, a segmentation Dice score of 95.89%, and a classification accuracy of 99.16%. The results support the possibility of real-world clinical use in early breast cancer diagnosis.

Gudur et al. (2024) [97] presented an advanced DL model for breast cancer detection based on mammograms. The model consists of CNNs, ResNet50, and recurrent neural networks (RNNs). The system includes image pre-processing, segmentation, and advanced feature extraction using ResNet50. Temporal pattern modeling is performed using RNNs. The RSNA mammogram database was used for modeling. The proposed method showed the accuracy of ResNet50, which reached 98%, which was superior to the architectures of CNNs and RNNs separately. The results of the study showed a decrease in the number of false positives and false negatives, which opens the potential for clinical application in early diagnostic processes.

The study by Mahmood et al. (2024) [98] presented a novel approach for breast cancer detection based on deep ML using multiparametric mammography. The proposed approach incorporates advanced preprocessing, radiomics, and hybrid neural network models (CNN + LSTM, CNN + SVM) for breast lesion detection, classification, and evaluation. The novelty of the model lies in the use of the Chaotic Leader Selective Filler Swarm Optimization (cLSFSO) algorithm for efficient mammogram segmentation, while transfer learning is achieved using modified VGGNet and SEResNet152 models. Publicly available databases were used to validate the system, and as a result, the system achieves high diagnostic accuracy (AUC = 0.99, sensitivity = 0.99). The authors of the study argue that the application of Grad-CAM improves interpretability by distinguishing significant regions, which may help in early and accurate detection of breast cancer.

The study by Muduli et al. (2020) [99] presented a computer-aided diagnosis (CAD) model combining the lifting wavelet transform (LWT), dimensionality reduction techniques (PCA and LDA), and extreme learning machines based on Moth Flame Optimization (MFO-ELM). The model is designed to detect breast cancer using digital mammograms. The system uses LWT to extract features from regions of interest (ROIs) in mammograms. Using PCA + LDA fusion strategies reduces the feature space, while ELM was used to classify mammograms. The proposed model achieved classification accuracy of 100% and 99.76% using MIAS and DDSM databases, respectively. The results of the study showed the effectiveness of the hybrid model, improving the performance and generalization ability of the systems.

In summary, it is evident from the reviewed works that the convergence of DL, optimization algorithms, and hybrid implementation methods is providing rapid progress in breast cancer diagnosis. To improve lesion detection and segmentation boundaries for interpretability and adaptation to clinical context, these systems develop a new system for early, accurate, and personalized breast cancer screening. Although the models maintain exceptional diagnostic accuracy, often exceeding 98–99%, the need for further validation of the best datasets in the world remains a common challenge to ensure wider dissemination of information and advancement in clinical practice.

### 7.2. Machine Learning, Deep Learning, and Hybrid Algorithms for Breast Cancer Detection Using Ultrasound Images

Numerous studies have been devoted to the use of ultrasound images for the diagnosis and classification of breast cancer. Table 3 presents some publications that apply ML, DL, and hybrid methods to analyze breast cancer using ultrasound images.

**Table 3:** Machine learning, deep learning, and hybrid techniques for breast cancer detection using ultrasound images.

Reference	ML, DL, Hybrid Algorithms	No. of Dataset Images	Performance Evaluation
Liu, Y.; Ren, L.; Cao, X.; Tong, Y. (2020) [100]	SVM with edge-based features (SMC, SMCP, and SMCSD)	192	*ACC* is 82.69%, *Sn* is 66.67%, *Sp* is 93.55%, *PPV* is 87.5%, *NPV* is 80.56%
Ametefe, D.S.; John, D.; Aliu, A.A.; Ametefe, G.D.; Hamid, A.; Darboe, T. (2025) [101]	Deep transfer learning (3 pre-trained CNNs: VGG16, VGG19, and EfficientNet) and U-Net	780	*ACC*: VGG16—95%, VGG19—95.5%, EfficientNetB3—85.8%; Sn: VGG16—94.1%, VGG19—94.06%, EfficientNetB3—86.8%; *Sp*: VGG16—96.6%, VGG19—97%, EfficientNetB3—85.3%; *Pr*: VGG16—96.4%, VGG19—96.9%, EfficientNetB3—85.8%; *F1*-score: VGG16—95.3%, VGG19—95.5%, EfficientNetB3—85.9%
Wang, C.; Guo, Y.; Chen, H.; Guo, Q.; He, H.; Chen, L.; Zhang, Q. (2025) [102]	GCN-based network, ABUS-Net	547	*AUC* is 82.6%; *ACC* is 86.4%; *Pr* is 92.6%; *Sn* is 71.4%; *Sp* is 96.2%; *F1*-score is 80.6%
Kiran, A.; Ramesh, J.V.N.; Rahat, I.S.; Khan, M.A.U.; Hossain, A.; Uddin, R. (2024) [103]	Hybrid approach: EfficientNetB3 and k-Nearest Neighbors	780	*ACC*, *Pr*, recall, and *F1*-score all at 100%
Tian, R.; Lu, G.; Tang, S.; Sang, L.; Ma, H.; Qian, W.; Yang, W. (2024) [104]	Transfer learning	1050	*ACC* is 0.964; *AUC* is 0.981

Liu et al. (2020) [100] proposed a novel ultrasound image feature extraction algorithm that combines edge-based features and morphological feature information. They mainly learn three features, including the sum of maximum curvature, the sum of maximum curvature and peak, and the sum of maximum curvature and standard deviation, according to the shape histogram of ultrasound breast tumors from a local perspective. Based on the results of the SVM classifier, this study showed a higher classification accuracy (82.69%). As the authors conclude, the inclusion of morphological features such as roughness, regularity, aspect ratio, ellipticity, and roundness leads to better recognition performance of the system compared with the control group, whose input only contained morphological features. Overall, the results of their research demonstrate the potential of edge-based features to better describe breast tumors in ultrasound images and to be used in computer-aided design of breast ultrasound examinations.

Ametefe et al. (2025) [101] explored the integration of deep transfer learning for classification and U-Net for segmentation to improve breast cancer detection using ultrasound imaging. Specifically, the deep transfer learning framework was implemented using the following pre-trained CNNs, such as VGG16, VGG19, and EfficientNet, as these models have strong capabilities for extracting the features. Meanwhile, the authors used the U-Net model to detect tumor boundaries with high spatial accuracy due to its effectiveness in medical image segmentation. The dataset used to evaluate the model includes ultrasound images classified as normal, benign, and malignant. According to the study results, VGG19 performed the best among other CNN models in terms of accuracy (95.5%), reproducibility (96.9%), specificity (97%), and *F1*-score (95.5%). U-Net also demonstrated its effectiveness in segmenting tumor regions across different lesion types. The average Dice similarity coefficient was 85.97%. Among the limitations, the authors noted high computational requirements, class imbalance, and insufficient diversity of datasets. Despite these challenges, the authors argue that AI-based models offer a robust diagnostic pipeline that improves lesion localization, reduces inter-observer variability, and facilitates clinical decision making.

Wang et al. (2025) [102] presented the ABUS-Net DL method for breast cancer diagnosis. This is a new GCN-based model (graph convolutional network) which uses Automated Breast Ultrasound (ABUS). Compared to traditional 3D patch-based methods, the ABUS-Net exploits coronal plane features and models the spatial relations between tumor slices using a graph-based structure. A multi-scale feature extraction module, which is built on ResNet50, enriches the tumor representation across different image scales. Analysis on private and public datasets demonstrated high performance, with accuracy, sensitivity, and specificity reaching 96.6%, and an *F1*-score of 94.9%. Thus, this research highlights the promise of graph-based multi-scale architecture in ABUS image analysis, suggesting a direction for improving structural modeling and classification accuracy in automated breast cancer detection.

Kiran et al. (2024) [103] introduced the efficient kNN model, which is considered a new hybrid DL approach. It integrates the EfficientNetB3 CNN with the k-Nearest Neighbors (kNN) classifier to enhance breast ultrasound image classification. The model, developed to detect benign, malignant, and normal cases, uses EfficientNetB3 for feature extraction and applies PCA for dimensionality reduction, followed by optimized k-NN classification. Efficient kNN, trained on a curated ultrasound dataset with extensive augmentation and preprocessing, outperformed traditional models, including VGG16, AlexNet, and VGG19 in diagnostic accuracy, precision, recall, and *F1*-score up to 100%. The research highlights the clinical potential of the model. In particular, the study emphasizes its interpretability, efficiency, and scalability for practical applications in healthcare.

In the study by Tian et al. (2024) [104], a hybrid diagnostic model combining traditional radiomics and DL was proposed to classify benign and malignant breast tumors in ultrasound images. The authors extracted radiomics features and deep features from several pretrained CNN models, including MNASNet, using a dataset of 1050 annotated cases. In order to improve diagnostic accuracy, they used feature fusion and different ML classifiers (SVM, XGBoost, LightGBM, etc.). The ensemble approach merging MNASNet features with radiomics reached a balanced accuracy of 0.964 and an AUC of 0.981. Multi-center validation, consuming external datasets, showed the generalizability of the model. The research emphasizes the potential of combining handcrafted and deep features for reliable, interpretable, and clinically meaningful detection of breast cancer.

In conclusion, the studies reviewed confirm that AI-enabled ultrasound imaging offers very promising opportunities for accurate, efficient, and interpretable breast cancer diagnosis. In addition to enhancements of diagnostic accuracy and reduction of human variability, in the future, these systems will also lay the foundation for smart imaging platforms tailored for various patient populations and medical settings.

### 7.3. Machine Learning, Deep Learning, and Hybrid Algorithms for Breast Cancer Detection Using Thermograms

Various studies are devoted to the use of thermography for breast cancer detection and classification. Table 4 presents some publications that used ML, DL, and hybrid methods for breast cancer detection in the analysis of thermograms.

**Table 4:** Machine learning, deep learning, and hybrid techniques for breast cancer detection using thermograms.

Reference	ML, DL, Hybrid Algorithms	Dataset	Performance Evaluation
Ekici, S.; Jawzal, H. (2020) [105]	CNN	Mastology research dataset	*ACC* is 98.95%
de Freitas Barbosa, V.A.; de Santana, M.A.; Andrade, M.K.S.; de Lima, R.d. C.F.; dos Santos, W.P. (2020) [106]	DWAN	Mastology research dataset	*Sn* is 0.95
Cabıoğlu, Ç.; and Oğul, H. (2020) [107]	CNN	DMR	*ACC* is 94.3%
Sánchez-Ruiz, D.; Olmos-Pineda, I.; Olvera-López, J.A. (2020) [108]	ROI ANN	Mastology research dataset	*ACC* is 90.2% *Sn* is 89.34% *Sp* is 91%
Resmini, R.; da Silva, L.F.; Medeiros, P.R.T.; Araujo, A.S.; Muchaluat-Saade, D.C.; Conci, A. (2021) [109]	K-star; SVM	DMR	*ACC* is 94.61%; *AUC* is 94.87%
Allugunti, V.R. (2022) [110]	CNN, SVM, RF	1000 images (from Kaggle)	*ACC*: CNN is 99.67%; SVM is 89.84%; RF is 90.55%
Mohamed, E.A.; Rashed, E.A.; Gaber, T.; Karam, O. (2022) [111]	CNN U_NET	DMR_IR	*ACC* is 99.3% *Sn* is 100% *Sp* is 98.67%
Civilibal, S.; Cevik, K.K.; Bozkurt, A. (2023) [112]	R-CNN with transfer learning	76 images of women	*ACC* is 97%; *Pr* is 96.1%; recall is 1.0; *F1*-score is 0.98
Ramacharan, S.; Margala, M.; Shaik, A.; Chakrabarti, P.; Chakrabarti, T. (2024) [113]	HERA-Net, integrating VGG19, U-Net, GRU, ResNet-50	DMR	*ACC* is 99.86; *Sn* is 100%; *Sp* is 99.81%

Ekici et al. (2020) [105] reviewed a new method for early detection of breast cancer using thermal imaging (thermography) combined with CNN. Considering limitations of traditional mammography, especially for young women with dense breast tissue, the authors offer a non-invasive, non-contact, and non-radiation method that exploits the distinct thermal patterns associated with malignant tissues. A CNN model optimized by Bayesian algorithms was developed in MATLAB and trained on a dataset that included 3895 thermographic images from 140 patients. This model achieved a testing accuracy of 98.95%. The researchers conclude that thermography enhanced by a DL method such as CNN has great potential in breast cancer screening as a feasible alternative or complement to mammography.

Cabıoğlu et al. (2020) [106] investigated CAD techniques for breast cancer detection based on thermal imaging. A number of CNN architectures have been developed using the learning transfer method. The results show that models including pre-trained convolutional layers merged with newly trained fully connected layers provide higher performance compared to other arrangements. Specifically, this approach achieved 94.3% accuracy, 94.7% precision, and 93.3% recall.

Sánchez-Ruiz et al. (2020) [108] introduced an entirely automatic approach for region of interest (ROI) segmentation in thermal breast images and their classification using artificial neural networks (ANN). The proposed segmentation method integrates color intensity thresholding, local contrast enhancement, and statistical edge detection to define appropriate breast regions. The extraction of features is implemented using statistical features of first and second order from histograms, temperature matrices, and gray-level co-occurrence matrices (GLCMs). A genetic algorithm (GA) is used for optimization of the ANN hyperparameters, improving the classification quality. The system was tested on the basis of 175 static thermographic images acquired using DMR. The results show average accuracy of 90.17%, sensitivity of 89.33%, specificity of 91.00%, and best-case accuracy (with GA and optimized subsets) of 98.33%.

Resmini et al. (2021) [109] presented a hybrid computational approach that combines dynamic infrared thermography (DIT) and static infrared thermography (SIT), respectively, for screening and diagnosis of breast cancer. The DIT data and temperature time series from segmented breast regions are used in the screening phase. It employs k-means clustering, cluster validity indices (e.g., Strehl index), and, subsequently, classification using the K-Star algorithm, reaching an average accuracy of 98.57%. The diagnostic phase employs SIT images in which textural features based on GLCM, LTP, wavelets, and fractal dimensions are extracted and selected through a genetic algorithm (GA). Classification is performed using SVM, achieving a diagnostic accuracy of 94.61% and an AUC of 94.87%.

Allugunti (2020) [110] presented a computer-aided design (CAD) system that sorts breast thermographic images into normal, benign, and malignant classes using an advanced CNN and compares its performance with SVM and RF classifiers. This approach includes preprocessing (data augmentation, artifact removal), segmentation (region growing), and extraction of features, with CNNs performing end-to-end training while ordinary classifiers use only handcrafted features. The model was trained on more than 1000 thermal images retrieved from Kaggle. It achieved 99.65% classification accuracy with CNNs, outperforming SVM (89.84%) and RF (90.55%). The study shows a structured pipeline from input to classification, highlighting the use of thermographic imaging and DL for effective non-invasive breast cancer diagnosis.

Mohamed (2022) [111] proposed an entirely automatic framework that combines U-Net and a custom DL model for classifying breast thermal images. The research starts with segmentation of the breast region from the thermograms by the U-Net to remove irrelevant regions, including the neck and shoulders, which may serve as noise during categorization. Afterwards, to differentiate between normal and abnormal breast tissue, a two-class CNN was trained. The model was assessed on the DMR-IR dataset, which includes 1000 frontal thermographic images (500 normal, 500 abnormal), and reached 99.33% accuracy, 100% sensitivity, and 98.67% specificity. The framework incorporates image resizing for computational efficiency, automatic ROI segmentation, and classification using a nine-layer CNN architecture. The results of comparative analysis, which was conducted using pre-trained CNN models (e.g., VGG16, ResNet18) and traditional ML classifiers (e.g., SVM, KNN), demonstrated that the approach shows higher performance on the selected metrics.

Civilibal (2023) [112] presented a unified structure that employs Mask R-CNN for simultaneous detection, segmentation, and classification of breast lesions in thermographic images. Using transfer learning with ResNet-50 and ResNet-101 frameworks pre-trained on the COCO dataset, this approach processes thermal images by outlining bounding boxes and generating pixel-level masks to separate normal breast tissue from pathological one. The author used a dataset of publicly accessible thermal images collected from 56 women (19 healthy and 37 with tumors). The ResNet-50-based Mask R-CNN reached higher performance with a classification accuracy of 97.1%, mean average precision (mAP) of 0.921, and an overlap rate of 86.8% in segmentation on the test data. Generally, the study shows the effectiveness of a single DL model for end-to-end breast thermogram analysis across such tasks as detection, segmentation, and classification.

Ramacharan (2024) [113] presented HERA-Net, a hybrid DL-based architecture designed to improve breast cancer diagnosis using both thermographic and ultrasound imaging. This model integrates VGG19, U-Net, GRU, and ResNet-50, respectively, for deep feature extraction, segmentation, temporal analysis, and classification. The approach starts with preprocessing steps that include grayscale conversion, CLAHE, bilateral filtering, and NLMS filtering for image quality enhancement. The local binary patterns (LBP) and histogram of oriented gradients (HOG) are used for feature extraction. This is followed by optimization using recursive feature elimination and cross-validation. The system was trained and tested on a dataset of 3534 thermographic images from the DMR database and reached 99.86% accuracy, 100% sensitivity, and 99.81% specificity.

Thus, AI-based thermographic imaging for breast cancer diagnosis has achieved technical maturity, providing superior diagnostic accuracy (often >97%) and promising the potential for scalable, real-time, and patient-friendly screening alternatives. Although further validation on diverse practical datasets is crucial for widespread clinical implementation, the convergence of thermography and DL represents a promising path toward earlier, safer, and more accessible breast cancer detection.

### 7.4. Multimodal Large Language Models, Large Language Models for Breast Cancer Diagnosis

MLLMs are increasingly being explored for breast cancer diagnosis due to their capacity for integration and analysis of various types of data, including genomic data, pathology reports, clinical notes, and medical imaging such as mammography, ultrasound, and thermography. These models use DL to process and analyze different types of data, improving diagnostic accuracy and providing a personalized approach. Below is an overview of their application in breast cancer diagnosis, based on recent studies. The possibility of using MLLMs for improved image analysis is demonstrated by authors such as Guo et al. and Rao et al. [114,115].

Guo et al. (2024) [114] proposed KAMnet, which is a novel diagnostic framework designed to advance the classification of breast cancer through the integration of domain-specific clinical knowledge into DL. It employs contrast-enhanced ultrasound (CEUS) and B-mode ultrasound (B-US) videos, using temporal attention and feature fusion to increase diagnostic precision. There are three forms of prior knowledge, such as temporal attention, spatial focus, and multimodal interaction, incorporated by KAMnet through four integration strategies at the data, network, decision, and training levels. These strategies include Gaussian-based keyframe sampling, a feature fusion network, joint classification inference, and a spatial attention-guided loss function using empirical activation matrices (EAMs). The KAMnet achieved a sensitivity of 90.91%, an accuracy of 88.24%, and an AUC of 0.943 during the evaluation on a custom BCUMV dataset with 332 cases. The research highlights the value of incorporating clinical knowledge into neural architectures to advance diagnostic interpretation and performance, and recognizes the need for larger multi-center datasets and integration of additional clinical variables in future studies.

The study of Rao et al. (2023) [115] explored the potential of LLMs, specifically ChatGPT-3.5 and GPT-4, as medical decision support approaches for radiologic evaluations associated with BC screening and breast pain. The GPT-4 has achieved 98.4% and 77.7% accuracy on breast cancer screening SATA prompts and on breast pain, respectively. In comparison to GPT-4, GPT-3.5 demonstrated 88.9% and 58.3% accuracy on the same indicators, respectively. In particular, GPT-4 demonstrated higher clinical utility and restraint. Sometimes it recommends against imaging when not indicated. The results of the study highlight the growing potential of GPT-4 as an aid in decision-making processes in radiology imaging studies, although limitations such as hallucinations, lack of source attribution, and model opacity remain.

LLMs can be used to integrate clinical data by processing unstructured data from electronic health records (EHRs), pathology reports, and clinical notes to extract relevant information such as biomarkers or patient history. Models such as ChatGPT-4 and Gemini can provide some insights by combining genetic data, biomarkers, and patient history to recommend personalized diagnostic protocols [116,117,118,119].

Sorin et al. (2023) [118] examined the feasibility and performance of ChatGPT-3.5 as a clinical decision support approach in a multidisciplinary breast tumor board. Clinical descriptions of ten consecutive breast cancer patients were entered into the model, and ChatGPT treatment recommendations were compared with actual tumor board decisions. Chatbot responses were independently assessed by two senior radiologists across three criteria: summary, clinical recommendations, and explanation. ChatGPT recommendations agreed with tumor board recommendations in 70% of cases. Although summary and explanation achieved high agreement scores, clinical recommendations received slightly lower scores. This highlights the complexity of nuanced decision making. The research also identified important limitations specifically related to ChatGPT’s occasional omission of critical clinical details (e.g., HER2 status) and lack of reference to radiologists in team recommendations. There are also general limitations of LLMs regarding the potential for hallucination, data bias, and cybersecurity risks.

The study by Fatima-Zahrae Nakach et al. (2024) [119] offered a systematic literature review of 47 major studies published between 2018 and 2023. It examines the application of multimodal DL fusion methods to improve breast cancer classification by integrating different types of data, including radiological images, clinical records, and genomic information. It is highlighted that multimodal methods significantly improve prognostic performance compared to unimodal models, with most studies reporting accuracy above 80%. The authors recommended future research to expand ensemble learning, incorporate underused modalities, explore co-learning and translation-based fusion, and improve explainable AI (XAI) strategies to support clinical applicability.

An alternative method for using LLMs is answering guideline-based questions and assisting in clinical decision making by summarizing scientific literature and medical care guidelines.

For instance, Haver et al. (2023) [120] assessed the reliability and clinical validity of ChatGPT’s answers to common patient questions about breast cancer prevention and screening. They submitted to ChatGPT a set of 25 questions derived from expert clinical experience and BI-RADS guidelines three times. Then the answers were evaluated by three board-certified breast radiologists. ChatGPT’s responses were found to be appropriate in 88% of cases, unreliable in 8%, and inappropriate in 4%. Although the model performed well in providing general information on BI-RADS screening and interpretation, inconsistencies and outdated recommendations in some responses highlighted the need for human review. The authors conclude that ChatGPT may improve patient education; however, it highlights limitations in consistency, sensitivity of prompts, and adherence that need to be addressed before clinical implementation.

Inconsistent accuracy can be highlighted among the challenges and limitations of multimodal LLMs [64,121,122,123,124] as performance differs across modalities and tasks. For instance, GPT-4V demonstrated 35.2% accuracy in identifying pathologies for ultrasound images in comparison to 66.7% on X-rays. The next limitation is data sensitivity and bias, as LLMs trained on biased or limited datasets may perpetuate healthcare inequalities, necessitating careful data curation. Ethical and regulatory concerns are the third limitation, whereas issues such as accountability, data privacy, and lack of standardized protocols limit clinical adoption. Finally, the sensitivity of prompts is also challenged as LLM performance depends on query formulation, and they may miss critical clinical details, requiring human supervision.

Future research should be focused on better integration of imaging, genomics, and clinical data to improve diagnostic accuracy. Additionally, LLM-specific reporting standards should be developed to ensure data comparability and reliability in clinical settings. Additionally, interpretability should be improved through methods such as human-in-the-loop validation to align model output with medical knowledge. Finally, oncology-specific adaptations of SLMs or domain-specific LLMs (e.g., Med-PaLM M) should be developed to address data safety and explainability issues.

In summary, multimodal LLMs have transformative potential in breast cancer diagnostics by integrating multiple data sources, improving early detection, and supporting personalized care. However, their limitations, like inconsistent accuracy, ethical concerns, and the need for validation, highlight the importance of careful integration under radiologist supervision. Continued improvements in model design, data quality, and regulatory frameworks will be critical to their reliability in practical use.

## 8. Discussion

Relatively recent advances in imaging-based medical diagnostics have significantly changed the approach to breast cancer diagnosis, especially through imaging modalities such as mammography, ultrasound, and thermography. This study provides a varied perspective on technological progress and clinical applicability by analyzing both traditional ML and DL approaches, as well as emerging directions in explainable AI (XAI) and LLMs.

Recent research on the application of AI to medical imaging, particularly when combined with DL techniques, has shown remarkable potential for automating and enhancing critical tasks such as breast cancer detection, segmentation, and classification. Studies employing traditional ML, DL, or hybrid algorithms have reported high performance levels, often exceeding 95%, indicating that AI technologies are approaching readiness for integration into clinical workflows. However, several methodological, practical, and infrastructural challenges remain, and addressing these issues is essential for the widespread adoption of AI in clinical settings.

In this review, we examine three types of medical imaging, mammography, ultrasound, and thermography, and analyze recent studies that combine these modalities with AI methods. Mammography remains the most widely used and extensively studied imaging modality for breast cancer detection. The availability of large, publicly accessible datasets such as MIAS, DDSM, INbreast, and CBIS-DDSM has facilitated the development and benchmarking of ML and DL algorithms. In mammography, AI models have evolved from traditional classifiers to advanced, highly integrated systems incorporating multi-scale learning, transformer architectures, and ensemble algorithms. Numerous studies report sensitivity and specificity exceeding 90%, with DL models (particularly CNN) outperforming traditional feature-based classifiers. In addition, a notable example is the large-scale MASAI study [91], which empirically demonstrates the benefits of these advances, including higher cancer detection rates and reductions in both false positives and false negatives.

Ultrasound-based studies have employed pre-trained DNN (e.g., EfficientNet, VGG19), often in combination with radiomics and network-based segmentation, achieving high sensitivity and specificity even in cases of dense breast tissue [100,101,102,103,104,105]. Multiple studies, including those by Shao et al. [38] and Zanello et al. [40], demonstrated improved cancer detection rates when ultrasound is combined with mammography. For example, Shao et al. showed a sensitivity improvement of 12% and an additional 1.9–3.5 cancers detected per 1000 women in dense breast populations. AI models applied to ultrasound data have achieved high accuracy, with some studies reporting sensitivity close to 100%. Algorithms such as CNNs, random forests, and SVMs have been employed for lesion classification, while segmentation tasks are frequently addressed using U-Net or Mask R-CNN architectures.

Thermography provides a non-invasive, radiation-free, and low-cost imaging alternative, making it particularly attractive for use in remote or resource-constrained regions. Its ability to detect physiological changes, such as increased vascularity and heat emission associated with tumor growth, offers a complementary perspective to anatomical imaging. It, though historically underutilized, has gained renewed interest due to its compatibility with DL classifiers. End-to-end thermogram-based systems such as Mask R-CNN and HERA-Net have achieved diagnostic accuracies exceeding 97%, particularly when incorporating dynamic thermograms or hybrid feature extraction algorithms [106,107,108,109,110,111,112,113,114]. Although improvements in image processing and the application of DL models have enhanced performance, thermography is not yet widely adopted as a standalone diagnostic tool.

Traditional ML techniques such as LR, SVMs, RF, and XGBoost have been used for feature selection, predictive modeling, and classification tasks. These approaches heavily rely on handcrafted features, which limit generalizability but allow transparency and interpretability. DL methods, particularly CNNs, have revolutionized medical image analysis by eliminating the need for manual feature engineering. Studies applying CNNs to mammograms and ultrasounds consistently demonstrate superior performance compared to traditional methods, particularly in detecting subtle features and complex patterns. Hybrid approaches, integrating DL with optimization algorithms or ensemble ML methods, have further improved robustness and diagnostic accuracy.

While some studies in this review employ legacy architectures such as VGGNet, particularly in recent works that use them as baseline models, it is important to note that the current state-of-the-art in medical imaging has advanced toward more powerful and efficient architectures. Recent approaches leverage Vision Transformers (ViT), Swin Transformers, EfficientNetV2, ConvNeXt, and foundation models like SAM (Segment Anything Model), which demonstrate superior performance in segmentation, classification, and generalization. These models provide better accuracy, faster convergence, and adaptability across imaging modalities. Highlighting this evolution is essential for directing future research toward more impactful AI solutions in clinical practice.

Despite the high performance of traditional ML or DL models, or their hybrid algorithms, there are certain limitations that prevent AI from being used in clinical practice. The most significant of these is the dependence of model performance on large, high-quality labeled databases, which is problematic in medical imaging due to the lack of stored data and the privacy of patient data. DL models require large amounts of labeled data to produce reliable results, but many of the studies cited were based on small or homogeneous prepared datasets. This deficiency of data not only produces the risk of overfitting but also bounds the generalizability of the obtained results.

As AI models become increasingly accurate, their interpretability has emerged as a central concern for clinical adoption. XAI tools such as Grad-CAM, LRP, LIME, and SHAP provide visual or feature-level explanations of predictions. In breast imaging, heatmaps highlighting suspicious regions align with radiological practice, but issues such as saliency map instability and variability across models raise questions about reliability. For clinicians, explanations are essential not only to validate AI outputs but also to detect biases or errors in the training data. Without interpretability, even highly accurate models may face resistance in regulated clinical environments where transparency and accountability are mandatory.

Another noteworthy drawback is the lack of standardized benchmarks and evaluation protocols. This limitation hinders the comparison and reproducibility of results across studies, ultimately restricting the generalization of findings. The limited methodological flexibility contributes to this issue, as models are often developed using narrow datasets without the necessary K-fold validation on external or collaborative cohorts.

In addition to the abovementioned limitations, supervised learning methods excel in most current studies, highlighting a gap in the methodology of AI methods. Such methods produce high model performance when large amounts of categorized data are available, but their performance declines when data is limited. Unsupervised and semi-supervised learning methods, as well as reinforcement learning paradigms, remain insufficiently exploited. These substitute learning methods can be of great value in developing AI models that are more adaptive, perform well when limited data are available, and can learn under feeble supervision. Regarding data availability, we should mention the diversity in terms of scanner types, lighting environments, image resolutions, and anatomical presentation. All these lead to inconsistency of the data and make AI models highly adaptive to the input data.

In summary, although AI-enabled imaging systems across all three modalities demonstrate high diagnostic potential, significant drawbacks limit AI methods for clinical application. These include an insufficient number of datasets, lack of standardized protocols for evaluating AI system performance, insufficient use of advanced learning paradigms, and lack of homogeneity of image variability. Forthcoming studies should prioritize the creation of multicenter, high-quality datasets; investigation of hybrid and unsupervised learning methods; and development of understandable, adaptive AI systems that can share data across modalities and settings. Ultimately, advances in breast cancer diagnostic research will not only rely on algorithmic innovations but also on the careful implementation of these systems within the complex ecosystem of modern medical practice.

## 9. Conclusions

This study reviews recent advances in breast cancer detection and classification using AI techniques applied to mammography, ultrasound, and thermography. Each modality offers distinct advantages: mammography remains the cornerstone of screening with extensive datasets and proven efficacy for early detection; ultrasound is particularly valuable for younger women and those with dense breast tissue; and thermography provides a non-invasive, radiation-free, and cost-effective adjunct that may improve access in resource-limited regions. Traditional ML methods have contributed significantly to tasks such as feature extraction, classification, and predictive modeling. AI models—particularly CNNs and hybrid algorithms—have markedly enhanced diagnostic accuracy and efficiency across these imaging modalities. The integration of explainable AI (XAI) improves interpretability and fosters clinical trust, while the emergence of LLMs such as GPT-4, Gemini, and LLaMA opens new opportunities for early detection, patient engagement, and decision support. Although several AI systems for breast cancer diagnosis—such as Transpara, ProFound AI, and Lunit INSIGHT—are already in clinical use and have demonstrated improvements in detection accuracy and workflow efficiency, they often remain limited to specific imaging modalities and face challenges related to transparency, interpretability, and integration across diverse healthcare settings. In addition, challenges remain in terms of dataset availability and diversity, model generalizability, reproducibility, and clinical integration. Explainable AI (XAI) is critical to enhance transparency and trust, yet current tools face limitations such as saliency instability. Looking ahead, LLMs and multimodal LLMs hold promises for integrating imaging data with clinical narratives and medical records, but issues of hallucination, governance, and auditability must be addressed. Although challenges remain regarding data quality, generalizability, and model transparency, these technologies demonstrate significant potential. Translating these advances into routine clinical practice requires not only technical improvements but also rigorous validation, ethical oversight, and collaboration between clinicians and researchers. Thus, ongoing research is crucial to ensure their ethical, explainable, and clinically effective adoption in real-world breast cancer diagnostics.

## Figures and Tables

**Table 1:** List of accessible datasets along with their respective URLs for reference.

Dataset Name	Image Modality	URL
Mammography Dataset		
MIAS	Mammogram	https://www.repository.cam.ac.uk/handle/1810/250394 (accessed on 3 January 2025)
mini-MIAS	Mammogram	http://peipa.essex.ac.uk/info/mias.html (accessed on 3 January 2025)
BCDR	Mammogram	https://bcdr.ceta-ciemat.es/information/about (accessed on 3 January 2025)
DDSM	Mammogram	https://www.kaggle.com/datasets/skooch/ddsm-mammography (accessed on 11 October 2025)
INBreast	Mammogram	https://biokeanos.com/source/INBreast (accessed on 11 October 2025)
WBCD	Multimodality	https://archive.ics.uci.edu/ml/datasets/Breast+Cancer+Wisconsin+(Diagnostic) (accessed on 3 January 2025)
WDBC	Multimodality	http://networkrepository.com/breast-cancer-wisconsin-wdbc.php (accessed on 3 January 2025)
IRMA	Mammogram	https://data.world/datasets/irma (accessed on 3 January 2025)
Breast-Cancer-Screening-DBT	Mammogram	https://www.cancerimagingarchive.net/collection/breast-cancer-screening-dbt/ (accessed on 11 October 2025)
CMMD	Mammogram	https://www.cancerimagingarchive.net/collection/cmmd/ (accessed on 11 October 2025)
Breast Micro-Calcifications Dataset	Mammogram	https://data.europa.eu/data/datasets/oai-zenodo-org-5036062?locale=en (accessed on 3 January 2025)
OPTIMAM Mammography Image Database	Mammogram	https://medphys.royalsurrey.nhs.uk/omidb/ (accessed on 3 January 2025)
Digital Mammography Dataset for Breast Cancer Diagnosis Research (DMID)	Mammogram	https://figshare.com/articles/dataset/_b_Digital_mammography_Dataset_for_Breast_Cancer_Diagnosis_Research_DMID_b_DMID_rar/24522883/1 (accessed on 3 January 2025)
Mammographic Mass—UCI Machine Learning Repository	Mammogram	https://archive.ics.uci.edu/dataset/161/mammographic+mass (accessed on 3 January 2025)
ICTRE—The Cancer Imaging Archive (TCIA)	Mammogram	https://www.cancerimagingarchive.net/collection/victre/ (accessed on 3 January 2025)
Ambra UNIFESP Mammography	Mammogram	https://mamografiaunifesp.ambrahealth.com/ (accessed on 3 January 2025)
CSAW-S	Mammogram	https://zenodo.org/records/4030660 (accessed on 11 October 2025)
Safe Haven (DaSH)	Mammogram	https://www.abdn.ac.uk/research/digital/platforms/safe-haven-dash/accessing-data/available-datasets/ (accessed on 3 January 2025)
Ultrasound Dataset		
BUSI	Ultrasound	https://www.kaggle.com/datasets/aryashah2k/breast-ultrasound-images-dataset (accessed on 3 January 2025)
OASBUD	Ultrasound	https://zenodo.org/records/545928#.X0xKf8hKg2z (accessed on 3 January 2025)
UDIAT	Ultrasound	upon request (accessed on 3 January 2025)
BrEaST	Ultrasound	https://www.cancerimagingarchive.net/collection/breast-lesions-usg/?utm_source (accessed on 3 January 2025)
QAMEBI	Ultrasound	https://qamebi.com/breast-ultrasound-images-database/ (accessed on 3 January 2025)
BUS-BRA	Ultrasound	https://www.kaggle.com/datasets/orvile/bus-bra-a-breast-ultrasound-dataset (accessed on 3 January 2025)
Thermography Dataset		
The Mastology Research with Infrared Image (DMR-IR)	thermograms	https://www.kaggle.com/datasets/asdeepak/thermal-images-for-breast-cancer-diagnosis-dmrir (accessed on 11 October 2025)
NIRAMAI Health Analytix	thermograms	https://niramai.com/about/thermalytix/ (accessed on 3 January 2025)

## Data Availability

All the data used in this study are presented within the manuscript. The links to the relevant datasets are properly formatted and included in the table, and the search and inclusion methodology is thoroughly described in Section 5.

## References

[1] Latha H.P., Ravi S., Saranya A. (2024). Breast Cancer Detection Using Machine Learning in Medical Imaging–A Survey. Procedia Comput. Sci..

[2] National Comprehensive Cancer Network (2025). NCCN Clinical Practice Guidelines in Oncology: Breast Cancer Screening and Diagnosis, Version 2. https://www.nccn.org/professionals/physician_gls/pdf/breast-screening.pdf.

[3] World Health Organization (2023). Breast Cancer: Screening and Early Detection.

[4] Gradishar W.J., Anderson B.O., Abraham J., Aft R., Agnese D., Allison K.H., Blair S.L., Burstein H.J., Dang C., Elias A.D. (2020). Breast cancer, version 3.2020, NCCN clinical practice guidelines in oncology. J. Compr. Cancer Netw..

[5] Bevers T.B., Niell B.L., Baker J.L., Bennett D.L., Bonaccio E., Camp M.S., Chikarmane S., Conant E.F., Eghtedari M., Flanagan M.R. (2023). NCCN Guidelines® Insights: Breast Cancer Screening and Diagnosis, Version 1.2023. J. Natl. Compr. Canc. Netw..

[6] Oeffinger K.C., Fontham E.T.H., Etzioni R., Herzig A., Michaelson J.S., Shih Y.-C.T., Walter L.C., Church T.R., Flowers C.R., LaMonte S.J. (2015). Breast cancer screening for women at average risk: 2015 guideline update from the american cancer society. JAMA.

[7] Cardoso F., Kyriakides S., Ohno S., Penault-Llorca F., Poortmans P., Rubio I.T., Zackrisson S., Senkus E., ESMO Guidelines Committee (2019). Early breast cancer: ESMO Clinical Practice Guidelines for diagnosis, treatment and follow-up†. Ann. Oncol..

[8] Lee C.H., Dershaw D.D., Kopans D., Evans P., Monsees B., Monticciolo D., Brenner R.J., Bassett L., Berg W., Feig S. (2010). Breast cancer screening with imaging: Recommendations from the Society of Breast Imaging and the ACR on the use of mammography, breast MRI, breast ultrasound, and other technologies for the detection of clinically occult breast cancer. J. Am. Coll. Radiol..

[9] Siu A.L., U.S. Preventive Services Task Force (2016). Screening for breast cancer: U.S. Preventive services task force recommendation statement. Ann. Intern. Med..

[10] Tagliafico A.S., Piana M., Schenone D., Lai R., Massone A.M., Houssami N. (2020). Overview of radiomics in breast cancer diagnosis and prognostication. Breast.

[11] Baltzer P.A.T., Kapetas P., Marino M.A., Clauser P. (2017). New diagnostic tools for breast cancer. Memo.

[12] Borgstede J.P., Bagrosky B.M. (2011). Screening of High-risk Patients. Early Diagnosis and Treatment of Cancer Series: Breast Cancer.

[13] Lowry K.P., Trentham-Dietz A., Schechter C.B., Alagoz O., Barlow W.E., Burnside E.S., Conant E.F., Hampton J.M., Huang H., Kerlikowske K. (2020). Long-term outcomes and cost-effectiveness of breast Cancer Screening with digital breast tomosynthesis in the United States. J. Natl. Cancer Inst..

[14] Løberg M., Lousdal M.L., Bretthauer M., Kalager M. (2015). Benefits and harms of mammography screening. Breast Cancer Res..

[15] Obeagu E.I., Obeagu G.U. (2024). Breast cancer: A review of risk factors and diagnosis. Medicine.

[16] Kennedy D.A., Lee T., Seely D. (2009). A comparative review of thermography as a breast cancer screening technique. Integr. Cancer Ther..

[17] USF Digital Mammography Home Page. http://www.eng.usf.edu/cvprg/Mammography/Database.html.

[18] DDSM Mammography. https://www.kaggle.com/datasets/skooch/ddsm-mammography.

[19] Moreira I.C., Amaral I., Domingues I., Cardoso A., Cardoso M.J., Cardoso J.S. (2012). INbreast: Toward a full-field digital mammographic database. Acad. Radiol..

[20] Suckling J. The Mini-MIAS Database of Mammograms. http://peipa.essex.ac.uk/info/mias.html.

[21] Breast Cancer Digital Repository. https://bcdr.ceta-ciemat.es/information/about.

[22] BREAST-CANCER-SCREENING-DBT-The Cancer Imaging Archive (TCIA). https://www.cancerimagingarchive.net/collection/breast-cancer-screening-dbt/.

[23] CMMD-The Cancer Imaging Archive (TCIA). https://www.cancerimagingarchive.net/collection/cmmd/.

[24] Loizidou K., Skouroumouni G., Pitris C., Pitris C. (2021). Breast Micro-calcifications Dataset with Precisely Annotated Sequential Mammograms. Eur. Radiol. Exp..

[25] Halling-Brown M.D., Warren L.M., Ward D., Lewis E., Mackenzie A., Wallis M.G., Wilkinson L.S., Given-Wilson R.M., McAvinchey R., Young K.C. (2021). OPTIMAM mammography image database: A large-scale resource of mammography images and clinical data. Radiol. Artif. Intell..

[26] CDD-CESM-The Cancer Imaging Archive (TCIA). https://www.cancerimagingarchive.net/collection/cdd-cesm/.

[27] Shin S.Y., Lee S., Yun I.D., Jung H.Y., Heo Y.S., Kim S.M., Lee K.M. (2015). A novel cascade classifier for automatic microcalcification detection. PLoS ONE.

[28] Digital Mammography Dataset for Breast Cancer Diagnosis Research (DMID) DMID.rar. https://figshare.com/articles/dataset/_b_Digital_mammography_Dataset_for_Breast_Cancer_Diagnosis_Research_DMID_b_DMID_rar/24522883/1.

[29] VinDr-Mammo: A Large-Scale Benchmark Dataset for Computer-Aided Detection and Diagnosis in Full-Field Digital Mammography v1.0.0. https://physionet.org/content/vindr-mammo/1.0.0/images/#files-panel.

[30] Mammographic Mass-UCI Machine Learning Repository. https://archive.ics.uci.edu/dataset/161/mammographic+mass.

[31] VICTRE-The Cancer Imaging Archive (TCIA). https://www.cancerimagingarchive.net/collection/victre/.

[32] Chen X., Zhang K., Abdoli N., Gilley P.W., Wang X., Liu H., Zheng B., Qiu Y. (2022). Transformers improve breast cancer diagnosis from unregistered multi-view mammograms. Diagnostics.

[33] Ambra|Home. https://mamografiaunifesp.ambrahealth.com/.

[34] Matsoukas C., Hernandez A.B.I., Liu Y., Dembrower K., Miranda G., Konuk E., Fredin Haslum J., Zouzos A., Lindholm P., Strand F. (2020). CSAW-S. Proceedings of the International Conference on Machine Learning (ICML).

[35] Safe Haven (DaSH)|Research|The University of Aberdeen. https://www.abdn.ac.uk/research/digital-research/dash.php.

[36] Catalano O., Fusco R., Carriero S., Tamburrini S., Granata V. (2024). Ultrasound findings after breast cancer radiation therapy: Cutaneous, pleural, pulmonary, and cardiac changes. Korean J. Radiol..

[37] Kim Y.E., Cha J.H., Kim H.H., Shin H.J., Chae E.Y., Choi W.J. (2023). The accuracy of mammography, ultrasound, and magnetic resonance imaging for the measurement of invasive breast cancer with extensive intraductal components. Clin. Breast Cancer.

[38] Shao Y., Hashemi H.S., Gordon P., Warren L., Wang J., Rohling R., Salcudean S. (2022). Breast cancer detection using multimodal time series features from ultrasound shear wave absolute vibroelastography. IEEE J. Biomed. Health Inform..

[39] Geisel J., Raghu M., Hooley R. (2018). The role of ultrasound in breast cancer screening: The case for and against ultrasound. Semin. Ultrasound CT MR.

[40] Zanello P.A., Robim A.F.C., de Oliveira T.M.G., Junior J.E., de Andrade J.M., Monteiro C.R., Filho J.M.S., Carrara H.H.A., Muglia V.F. (2011). Breast ultrasound diagnostic performance and outcomes for mass lesions using Breast Imaging Reporting and Data System category 0 mammogram. Clinics.

[41] Al-Dhabyani W., Gomaa M., Khaled H., Fahmy A. (2020). Dataset of breast ultrasound images. Data Brief.

[42] Piotrzkowska-Wróblewska H., Dobruch-Sobczak K., Byra M., Nowicki A. (2017). Open access database of raw ultrasonic signals acquired from malignant and benign breast lesions. Med. Phys..

[43] UDIAT Diagnostic Centre, Parc Taulí Corporation, Sabadell, Spain. https://www.kaggle.com/datasets?search=udiat.

[44] Achiam J., Adler S., Agarwal S., Ahmad L., Akkaya I., Aleman F.L., Almeida D., Altenschmidt J., Altman S., OpenAI (2024). GPT 4 Technical Report. arXiv.

[45] Ardakani A.A., Mohammadi A., Mirza-Aghazadeh-Attari M., Acharya U.R. (2023). Acharya, An open-access breast lesion ultrasound image database: Applicable in artificial intelligence studies. Comput. Biol. Med..

[46] Hamyoon H., Chan W.Y., Mohammadi A., Kuzan T.Y., Mirza-Aghazadeh-Attari M., Leong W.L., Altintoprak K.M., Vijayananthan A., Rahmat K., Ab Mumin N. (2022). Artificial intelligence, BI-RADS evaluation and morphometry: A novel combination to diagnose breast cancer using ultrasonography, results from multi-center cohorts. Eur. J. Radiol..

[47] Homayoun H., Chan W.Y., Kuzan T.Y., Leong W.L., Altintoprak K.M., Mohammadi A., Vijayananthan A., Rahmat K., Leong S.S., Mirza-Aghazadeh-Attari M. (2022). Applications of machine-learning algorithms for prediction of benign and malignant breast lesions using ultrasound radiomics signatures: A multi-center study. Biocybern. Biomed. Eng..

[48] Gómez-Flores W., Gregorio-Calas M.J., Coelho de Albuquerque Pereira W. (2024). BUS-BRA: A breast ultrasound dataset for assessing computer-aided diagnosis systems. Med. Phys..

[49] Vreugdenburg T.D., Willis C.D., Mundy L., Hiller J.E. (2013). A systematic review of elastography, electrical impedance scanning, and digital infrared thermography for breast cancer screening and diagnosis. Breast Cancer Res. Treat..

[50] Lashkari A., Pak F., Firouzmand M. (2016). Full intelligent cancer classification of thermal breast images to assist physician in clinical diagnostic applications. J. Med. Signals Sens..

[51] FDA (2019). FDA Warns Thermography Should not Be Used in Place of Mammography to Detect, Diagnose, or Screen for Breast Cancer: FDA Safety Communication. US Food & Drug Administration. https://www.fda.gov/news-events/press-announcements/fda-issues-warning-letter-clinic-illegally-marketing-unapproved-thermography-device-warns-consumers.

[52] Mashekova A., Zhao Y., Ng E.Y., Zarikas V., Fok S.C., Mukhmetov O. (2022). Mukhmetov, Early detection of the breast cancer using infrared technology—A comprehensive review. Therm. Sci. Eng. Prog..

[53] Shen Y., Heacock L., Elias J., Hentel K.D., Reig B., Shih G., Moy L. (2023). ChatGPT and other large language models are double-edged swords for medical diagnosis. Nat. Med..

[54] Nath M.K., Sundararajan K., Mathivanan S., Thandapani B. (2025). Analysis of breast cancer classification and segmentation techniques: A comprehensive review. Inform. Med. Unlocked.

[55] Aerts H.J.W.L., Velazquez E.R., Leijenaar R.T.H., Parmar C., Grossmann P., Carvalho S., Bussink J., Monshouwer R., Haibe-Kains B., Rietveld D. (2014). Decoding tumour phenotype by noninvasive imaging using a quantitative radiomics approach. Nat. Commun..

[56] Castiglioni I., Gallivanone F., Soda P., Avanzo M., Stancanello J., Aiello M., Interlenghi M., Salvatore M. (2019). AI based applications in hybrid imaging: How to build smart and truly multiparametric decision models for radiomics. Eur. J. Nucl. Med. Mol. Imaging.

[57] Sala E., Mema E., Himoto Y., Veeraraghavan H., Brenton J., Snyder A., Weigelt B., Vargas H. (2017). Unravelling tumour heterogeneity using next-generation imaging: Radiomics, radiogenomics, and habitat imaging. Clin. Radiol..

[58] Lambin P., Leijenaar R.T.H., Deist T.M., Peerlings J., de Jong E.E.C., van Timmeren J., Sanduleanu S., Larue R.T.H.M., Even A.J.G., Jochems A. (2017). Radiomics: The bridge between medical imaging and personalized medicine. Nat. Rev. Clin. Oncol..

[59] Li Y., Wu F.-X., Ngom A. (2016). A review on machine learning principles for multi-view biological data integration. Brief. Bioinform..

[60] Litjens G., Kooi T., Bejnordi B.E., Setio A.A.A., Ciompi F., Ghafoorian M., van der Laak J.A.W.M., van Ginneken B., Sánchez C.I. (2017). A survey on deep learning in medical image analysis. Med. Image Anal..

[61] Willemink M.J., Koszek W.A., Hardell C., Wu J., Fleischmann D., Harvey H., Folio L.R., Summers R.M., Rubin D.L., Lungren M.P. (2020). Preparing medical imaging data for machine learning. Radiology.

[62] Sahiner B., Pezeshk A., Hadjiiski L.M., Wang X., Drukker K., Cha K.H., Summers R.M., Giger M.L. (2019). Deep learning in medical imaging and radiation therapy. Med. Phys..

[63] Castiglioni I., Rundo L., Codari M., Di Leo G., Salvatore C., Interlenghi M., Gallivanone F., Cozzi A., D’Amico N.C., Sardanelli F. (2021). AI applications to medical images: From machine learning to deep learning. Phys. Medica Eur. J. Med. Phys..

[64] Ghorbian M., Ghobaei-Arani M., Ghorbian S. (2025). Transforming breast cancer diagnosis and treatment with large language Models: A comprehensive survey. Methods.

[65] Singhal K., Azizi S., Tu T., Mahdavi S.S., Wei J., Chung H.W., Scales N., Tanwani A., Cole-Lewis H., Pfohl S. (2023). Large language models encode clinical knowledge. Nature.

[66] Sallam M. (2023). ChatGPT utility in healthcare education, research, and practice: Systematic review on the promising perspectives and valid concerns. Healthcare.

[67] Biswas S. (2023). Role of ChatGPT in public health. Ann. Biomed. Eng..

[68] Ribeiro M.T., Singh S., Guestrin C. “Why should I trust you?”: Explaining the predictions of any classifier. Proceedings of the 22nd ACM SIGKDD International Conference on Knowledge Discovery and Data Mining.

[69] Lundberg S.M., Lee S.-I. (2017). A unified approach to interpreting model predictions. Adv. Neural Inf. Process. Syst..

[70] Simonyan K., Vedaldi A., Zisserman A. (2013). Deep inside convolutional networks: Visualising image classification models and saliency maps. arXiv.

[71] Sundararajan M., Taly A., Yan Q. Axiomatic attribution for deep networks. Proceedings of the 34th International Conference on Machine Learning.

[72] Breiman L. (2001). Random forests. Mach. Learn..

[73] Fisher A., Rudin C., Dominici F. (2019). All models are wrong, but many are useful: Learning a variable’s importance by studying an entire class of prediction models simultaneously. J. Mach. Learn. Res..

[74] Wachter S., Mittelstadt B., Russell C. (2017). Counterfactual explanations without opening the black box: Automated decisions and the GDPR. Harv. J. Law Technol..

[75] Ribeiro M.T., Singh S., Guestrin C. Anchors: High-precision model-agnostic explanations. Proceedings of the 32nd AAAI Conference on Artificial Intelligence.

[76] Craven M.W., Shavlik J.W. (1996). Extracting tree-structured representations of trained networks. Adv. Neural Inf. Process. Syst..

[77] Zarikas V., Georgakopoulos S.V. Interpretable Medical Image Diagnosis Methodology using Convolutional Neural Networks and Bayesian Networks. Proceedings of the 2024 9th International Conference on Mathematics and Computers in Sciences and Industry (MCSI).

[78] Aidossov N., Zarikas V., Zhao Y., Mashekova A., Ng E.Y.K., Mukhmetov O., Mirasbekov Y., Omirbayev A. (2023). An Integrated Intelligent System for Breast Cancer Detection at Early Stages Using IR Images and Machine Learning Methods with Explainability. SN Comput. Sci..

[79] Aidossov N., Zarikas V., Mashekova A., Zhao Y., Ng E.Y.K., Midlenko A., Mukhmetov O. (2023). Evaluation of Integrated CNN, Transfer Learning, and BN with Thermography for Breast Cancer Detection. Appl. Sci..

[80] Omirbayev A., Aidossov N., Zarikas V., Mashekova A., Zhao Y., Ng Y.K. Breast cancer diagnosis using thermograms and Bayesian and Convolutional Neural Networks. Proceedings of the Iupesm World Congress on Medical Physics and Biomedical Engineering (IUPESM WC2022).

[81] Abdel-Nasser M., Rashwan H.A., Puig D., Moreno A. (2015). Analysis of tissue abnormality and breast density in mammographic images using a uniform local directional pattern. Expert Syst. Appl..

[82] Touvron H., Lavril T., Izacard G., Martinet X., Lachaux M.A., Lacroix T., Rozière B., Goyal N., Hambro E., Azhar F. (2023). LLaMA: Open and Efficient Foundation Language Models. arXiv.

[83] Alshuhri M.S., Al Musawi S.G., Al Alwany A.A., Uinarni H., Rasulova I., Rodrigues P., Alkhafaji A.T., Alshanberi A.M., Alawadi A.H., Abbas A.H. (2024). Artificial intelligence in cancer diagnosis: Opportunities and challenges. Pathol. Res. Pract..

[84] Khalighi S., Reddy K., Midya A., Pandav K.B., Madabhushi A., Abedalthagafi M. (2024). Artificial intelligence in neuro oncology: Advances and challenges in brain tumor diagnosis, prognosis, and precision treatment. NPJ Precis. Oncol..

[85] Bhayana R. (2024). Chatbots and Large Language Models in Radiology: A Practical Primer for Clinical and Research Applications. Radiology.

[86] Habchi Y., Kheddar H., Himeur Y., Belouchrani A., Serpedin E., Khelifi F., Chowdhury M.E.H. (2025). Advanced deep learning and large language models: Comprehensive insights for cancer detection. Image Vis. Comput..

[87] Sha Z., Hu L., Rouyendegh B.D. (2020). Deep learning and optimization algorithms for automatic breast cancer detection. Int. J. Imaging Syst. Technol..

[88] Sapate S., Talbar S., Mahajan A., Sable N., Desai S., Thakur M. (2020). Breast cancer diagnosis using abnormalities on ipsilateral views of digital mammograms. Biocybern. Biomed. Eng..

[89] Mansour S., Kamal R., Hussein S.A., Emara M., Kassab Y., Taha S.N., Gomaa M.M. (2025). Enhancing detection of previously missed non-palpable breast carcinomas through artificial intelligence. Eur. J. Radiol. Open.

[90] Malherbe K. (2025). Revolutionizing Breast Cancer Screening: Integrating Artificial Intelligence with Clinical Examination for Targeted Care in South Africa. J. Radiol. Nurs..

[91] Hernström V., Josefsson V., Sartor H., Schmidt D., Larsson A.-M., Hofvind S., Andersson I., Rosso A., Hagberg O., Lång K. (2025). Screening performance and characteristics of breast cancer detected in the Mammography Screening with Artificial Intelligence trial (MASAI): A randomised, controlled, parallel-group, non-inferiority, single-blinded, screening accuracy study. Lancet Digit. Health.

[92] Nour A., Boufama B. (2025). Hybrid deep learning and active contour approach for enhanced breast lesion segmentation and classification in mammograms. Intell.-Based Med..

[93] Umamaheswari T., Babu Y.M.M. (2024). ViT-MAENB7: An innovative breast cancer diagnosis model from 3D mammograms using advanced segmentation and classification process. Comput. Methods Programs Biomed..

[94] Mannarsamy V., Mahalingam P., Kalivarathan T., Amutha K., Paulraj R.K., Ramasamy S. (2025). Sift-BCD: SIFT-CNN integrated machine learning-based breast cancer detection. Biomed. Signal Process. Control.

[95] Puttegowda K., Veeraprathap V., Kumar H.S.R., Sudheesh K.V., Prabhavathi K., Vinayakumar R., Tabianan K. (2025). Enhanced Machine Learning Models for Accurate Breast Cancer Mammogram Classification. Glob. Transit..

[96] Ahmad J., Akram S., Jaffar A., Ali Z., Bhatti S.M., Ahmad A., Rehman S.U. (2024). Deep learning empowered breast cancer diagnosis: Advancements in detection and classification. PLoS ONE.

[97] Gudur R., Patil N., Thorat S.T. (2024). Early Detection of Breast Cancer using Deep Learning in Mammograms. J. Pioneer. Med. Sci..

[98] Mahmood T., Saba T., Rehman A., Alamri F.S. (2024). Harnessing the power of radiomics and deep learning for improved breast cancer diagnosis with multiparametric breast mammography. Expert Syst. Appl..

[99] Muduli D., Dash R., Majhi B. (2020). Automated breast cancer detection in digital mammograms: A moth flame optimization based ELM approach. Biomed. Signal Process. Control.

[100] Liu Y., Ren L., Cao X., Tong Y. (2020). Breast tumors recognition based on edge feature extraction using support vector machine. Biomed. Signal Process. Control.

[101] Ametefe D.S., John D., Aliu A.A., Ametefe G.D., Hamid A., Darboe T. (2025). Advancing breast cancer diagnosis: Integrating deep transfer learning and U-Net segmentation for precise classification and delineation of ultrasound images. Results Eng..

[102] Wang C., Guo Y., Chen H., Guo Q., He H., Chen L., Zhang Q. (2025). ABUS-Net: Graph convolutional network with multi-scale features for breast cancer diagnosis using automated breast ultrasound. Expert Syst. Appl..

[103] Kiran A., Ramesh J.V.N., Rahat I.S., Khan M.A.U., Hossain A., Uddin R. (2024). Advancing breast ultrasound diagnostics through hybrid deep learning models. Comput. Biol. Med..

[104] Tian R., Lu G., Tang S., Sang L., Ma H., Qian W., Yang W. (2024). Benign and malignant classification of breast tumor ultrasound images using conventional radiomics and transfer learning features: A multicenter retrospective study. Med. Eng. Phys..

[105] Ekici S., Jawzal H. (2020). Breast cancer diagnosis using thermography and convolutional neural networks. Med. Hypotheses.

[106] De Freitas Barbosa V.A., de Santana M.A., Andrade M.K.S., de Lima R.d.C.F., dos Santos W.P. (2020). Deep-wavelet neural networks for breast cancer early diagnosis using mammary thermographies. Deep Learning for Data Analytics.

[107] Cabıoğlu Ç., Oğul H. (2020). Computer-aided breast cancer diagnosis from thermal images using transfer learning. Proceedings of the International Work-Conference on Bioinformatics and Biomedical Engineering.

[108] Sánchez-Ruiz D., Olmos-Pineda I., Olvera-López J.A. (2020). Automatic region of interest segmentation for breast thermogram image classification. Pattern Recognit. Lett..

[109] Resmini R., da Silva L.F., Medeiros P.R., Araujo A.S., Muchaluat-Saade D.C., Conci A. (2021). A hybrid methodology for breast screening and cancer diagnosis using thermography. Comput. Biol. Med..

[110] Allugunti V.R. (2022). Breast cancer detection based on thermographic images using machine learning and deep learning algorithms. Int. J. Eng. Comput. Sci..

[111] Mohamed E.A., Rashed E.A., Gaber T., Karam O. (2022). Deep learning model for fully automated breast cancer detection system from thermograms. PLoS ONE.

[112] Civilibal S., Cevik K.K., Bozkurt A. (2023). A deep learning approach for automatic detection, segmentation and classification of breast lesions from thermal images. Expert Syst. Appl..

[113] Ramacharan S., Margala M., Shaik A., Chakrabarti P., Chakrabarti T. (2024). Advancing Breast Cancer Diagnosis: The Development and Validation of the HERA-Net Model for Thermographic Analysis. Comput. Mater. Contin..

[114] Guo D., Lu C., Chen D., Yuan J., Duan Q., Xue Z., Liu S., Huang Y. (2024). A multimodal breast cancer diagnosis method based on Knowledge-Augmented Deep Learning. Biomed. Signal Process. Control.

[115] Rao A., Kim J., Kamineni M., Pang M., Lie W., Dreyer K.J., Succi M.D. (2023). Evaluating GPT as an Adjunct for Radiologic Decision Making: GPT-4 Versus GPT-3.5 in a Breast Imaging Pilot. J. Am. Coll. Radiol..

[116] Miao S., Jia H., Cheng K., Hu X., Li J., Huang W., Wang R. (2022). Deep learning radiomics under multimodality explore association between muscle/fat and metastasis and survival in breast cancer patients. Brief. Bioinform..

[117] Choi H.S., Song J.Y., Shin K.H., Chang J.H., Jang B.-S. (2023). Developing prompts from large language model for extracting clinical information from pathology and ultrasound reports in breast cancer. Radiat. Oncol. J..

[118] Sorin V., Klang E., Sklair-Levy M., Cohen I., Zippel D.B., Lahat N.B., Konen E., Barash Y. (2023). Large language model (ChatGPT) as a support tool for breast tumor board. npj Breast Cancer.

[119] Nakach F.-Z., Idri A., Goceri E. (2024). A comprehensive investigation of multimodal deep learning fusion strategies for breast cancer classification. Artif. Intell. Rev..

[120] Haver H.L., Ambinder E.B., Bahl M., Oluyemi E.T., Jeudy J., Yi P.H. (2023). Appropriateness of Breast Cancer Prevention and Screening Recommendations Provided by ChatGPT. Radiology.

[121] Grigore I., Popa C.-A. (2024). MambaDepth: Enhancing Long-range Dependency for Self-Supervised Fine-Structured Monocular Depth Estimation. arXiv.

[122] Griewing S., Lechner F., Gremke N., Lukac S., Janni W., Wallwiener M., Wagner U., Hirsch M., Kuhn S. (2024). Proof-of-concept study of a small language model chatbot for breast cancer decision support–a transparent, source-controlled, explainable and data-secure approach. J. Cancer Res. Clin. Oncol..

[123] AlSaad R., Abd-alrazaq A., Boughorbel S., Ahmed A., Renault M.A., Damseh R., Sheikh J. (2024). Multimodal Large Language Models in Health Care: Applications, Challenges, and Future Outlook. J. Med. Internet Res..

[124] Sorin V., Glicksberg B.S., Artsi Y., Barash Y., Konen E., Nadkarni G.N., Klang E. (2024). Utilizing large language models in breast cancer management: Systematic review. J. Cancer Res. Clin. Oncol..

